# Caloric restriction mimetics improve gut microbiota: a promising neurotherapeutics approach for managing age-related neurodegenerative disorders

**DOI:** 10.1007/s10522-024-10128-4

**Published:** 2024-08-23

**Authors:** Ishika Singh, Shashi Anand, Deepashree J. Gowda, Amitha Kamath, Abhishek Kumar Singh

**Affiliations:** Manipal Centre for Biotherapeutics Research, Manipal Academy of Higher Education, Karnataka Manipal, 576 104 India

**Keywords:** Caloric restriction mimetics, Gut microbiota, Gut-brain axis, Aging, Neurodegenerative disorders

## Abstract

The gut microbiota (GM) produces various molecules that regulate the physiological functionality of the brain through the gut-brain axis (GBA). Studies suggest that alteration in GBA may lead to the onset and progression of various neurological dysfunctions. Moreover, aging is one of the prominent causes that contribute to the alteration of GBA. With age, GM undergoes a shift in population size and species of microflora leading to changes in their secreted metabolites. These changes also hamper communications among the HPA (hypothalamic–pituitary–adrenal), ENS (enteric nervous system), and ANS (autonomic nervous system). A therapeutic intervention that has recently gained attention in improving health and maintaining communication between the gut and the brain is calorie restriction (CR), which also plays a critical role in autophagy and neurogenesis processes. However, its strict regime and lifelong commitment pose challenges. The need is to produce similar beneficial effects of CR without having its rigorous compliance. This led to an exploration of calorie restriction mimetics (CRMs) which could mimic CR’s functions without limiting diet, providing long-term health benefits. CRMs ensure the efficient functioning of the GBA through gut bacteria and their metabolites i.e., short-chain fatty acids, bile acids, and neurotransmitters. This is particularly beneficial for elderly individuals, as the GM deteriorates with age and the body’s ability to digest the toxic accumulates declines. In this review, we have explored the beneficial effect of CRMs in extending lifespan by enhancing the beneficial bacteria and their effects on metabolite production, physiological conditions, and neurological dysfunctions including neurodegenerative disorders.

## Introduction

The capability of the gut to control overall body processes has drawn researchers' attention. Numerous microbes inhabit the gut, and their concentration grows from the ileum to the colon (Sender et al. 2016). Gut microbiota (GM) plays a significant role in activities like producing vitamins and other nutrients, facilitating digestion, gut motility, inhibiting the colonization of foreign pathogens in the gut, neurotransmitter release, synthesis of several metabolites and antioxidants, and bolstering the immune system (Lozupone et al. 2012). The body and brain are affected by the GM be it positively or negatively. There exists galore of proof for mechanisms and pathways explaining how GM and its metabolites affect brain development and function via connecting with the brain. The GM may control the brain through five main pathways of communication, including the GM metabolic system, neuronal networks, neuroendocrine system, gut immune system, and barrier system pathways (Wang and Wang 2016). There are two ways that the gut flora and the brain communicate with each other through neural networks. The autonomic nervous system (ANS) and the vagus nerve in the spinal cord form the first neuroanatomical pathway, which directly links the gut and brain (Bonaz et al. 2017). The enteric nervous system (ENS) in the gut, the ANS, and the vagus nerve in the spinal cord comprise the second neuroanatomical pathway, which facilitates bidirectional communication. An additional pathway for interaction between gut bacteria and the brain is via the hypothalamic–pituitary–adrenal (HPA) axis, a neuroendocrine system that controls the body's stress response (Sudo et al. 2004).

Age is one of the conditioning elements that determine the viability of colonization by microbes in the GI tract, which begins at birth and changes throughout life. It is a multifaceted phenomenon that declines molecular and cellular function causing structural deterioration i.e. white matter disintegration and reduction in gray matter in the medial prefrontal cortex (mPFC) region leading to a range of neurodegenerative disorders. Studies have proved the involvement of GM in aging processes (Verheggen et al. 2020).

The disorders of the CNS involve pathological processes that lead to a decline in neurons (Klempin and Kempermann 2007; Isaev et al. 2019). The neurons are generated through the process called neurogenesis in the lateral ventricles (subventricular zone) and the dentate gyrus (subgranular zone) in the hippocampus. It also includes the production of glial cells, neural stem cells (NSCs) derived neural lineages, and neural progenitor cells (NPCs) (Apple et al. 2017). The data lends credence to the theory that the gut microbiome regulates and initiates neurogenesis (Ribeiro et al. 2020). It is well documented that the GM creates microbial metabolites such as SCFA, acetate, butyrate, propionate, and tryptophan, and its precursor indoles and even neurotransmitters can influence hippocampus neuroplasticity and encourage the neuronal differentiation of hippocampus neural progenitor cells (Yang et al. 2020b). Any alteration in this proportion of gut bacteria will have an impact on the neurogenesis process (Liu et al. 2022). Changes in GM are known to regulate neurodegeneration by inducing mitochondrial dysfunction and senescence (MiDAS), making it a pathophysiological aspect in diseases such as AD, PD, etc. Moreover, senescent cells in the gut lead to inflammation and hinder the ability to restore the normal microflora. This disruption is transferred throughout the etiopathogenetic cluster pathways giving rise to systemic inflammation and oxidative stress (Homolak 2023a). Therefore, there are interventions i.e., antibiotics (Möhle et al. 2016), microbial metabolites (Yang et al. 2020b), prebiotic dietary interventions (Beltz et al. 2007; Borsini et al. 2020), intermediate fasting (Gabarró-Solanas et al. 2023), CR (Ma et al. 2021), fecal microbiota transplant (Kim et al. 2021) which presents good therapeutics staggery in preclinical studies to address aberrant neurogenesis, by modulating the GM which may contribute to some neurological diseases.

This review addresses one of the ‘Seven knowledge gaps in modern biogerontology’ (Rattan 2024), which is how the relationship between CRMs and gut microbiota helps compensate for the changes that develop as a result of aging. CRMs help alleviate aging-induced oxidative damage, and inflammation and reduce neurodegeneration, thus enhancing the quality of life and increasing lifespan.

## The gut microbiota

### Composition and function

It has been well established that the GM performs an integral part in the development of the host immune system, synthesis of essential metabolites as well as exerting its influence on the brain through the gut-brain axis (Borrego-Ruiz and Borrego 2024). The colonization of the gut initiates from birth with the infant's contact with the microbiota of the amniotic fluid and placenta (Hasan and Yang 2019). It has been found that the colonization of bacteria in the colon is the densest as compared to anywhere else, making the colon the most heavily populated part of the gut by bacteria (Rinninella et al. 2019). This GM is, however, not static but highly dynamic, evolving and adapting to the physiological changes in the host, aging being a crucial factor. The research has suggested that a healthy adult gut microbiota accounts for majorly 12 bacterial phyla out of which the prominent four phyla *Actinomycetota, Bacillota, Bacteroidota*, and *Pseudomonadota* account for more than 93%; prominent bacterial families include *Bacteroidaceae* (65.6%), *Lachnospiraceae* (11.5%), and *Ruminococcaceae* (8.4%) and the most abundant bacterial genera being *Bacteroides* (> 65%). Apart from that, bacterial genes account for 99% of the genes that have been characterized from the human genome (Borrego-Ruiz and Borrego 2024). The functional aspect of a healthy GM is associated with metabolizing nutrients, xenobiotics, and other drugs (Jandhyala et al. 2015). Apart from this GM also facilitates a check on pathogenic microbial strains by regulating the local immune response (Jandhyala et al. 2015).

### Factors influencing the gut microbiota

Age is one of the crucial factors that determines the composition of GM at distinct stages of life. The microbial colonization of the gut evolves and changes over time to become more distinct and comparatively more stable. By the time a child reaches 3 years of age, this GM starts resembling that of an adult. As an individual approaches old age, changes in the GM can even prove to be detrimental. During aging, the alteration in gut microbial diversity may disturb the production of microbial metabolites such as short-chain fatty acids (SCFA), and bile acids, as well as neurotransmitters and also inflammatory markers in the blood, decreasing the function of the blood–brain barrier (BBB), which as this BBB becomes more permeable with age (Verheggen et al. 2020), and loses its ability to influx the deleterious blood components leading to neuroinflammation (Tran and Mohajeri 2021) and accelerating towards various neurological disorders such as Alzheimer’s disease (AD), Parkinson’s disease (PD), schizophrenia, autism spectrum disorder (ASD), major depressive disorder (MDD), bipolar disorder (BD), and anxiety disorders (McGuinness et al. 2022; Ferreiro et al. 2023). It has also been found that there is a decline in lactate derived from GM with advancing age leading to disruption of lactate: pyruvate ratio. This disruption gives rise to a dysfunctional intestinal barrier. The same was proven by comparing aged rats and young rats, wherein aged rats having declined lactate production were prone to enhanced permeability of the intestine (Papila et al. 2024).

Another principal factor that helps determine the composition of GM is diet. The initial colonization of GM is dominated by *Lactobacillus* and *Bifidobacterium* found in dietary sources like breast milk. These microbes help in the expression of IgG antibodies resulting from the surge in short-chain fatty acid from the breakdown of oligosaccharides present in breast milk (Hasan and Yang 2019)*.* Western diet including food with high sugar, saturated fat, and animal protein content inclines a shift from *Bacteroidota* to increased *Pseudomonadota* and *Bacillota* members in the gut. On the other hand, a ‘Mediterranean diet’ composed of unsaturated fatty acids, fibers, and antioxidants has been shown to help in longevity by shifting the GM towards *Bacteroides, Bifidobacterium, and Lactobacillus,* with a reduction in the members of *Pseudomonadota* and *Bacillota* (Borrego-Ruiz and Borrego 2024). Moreover, antibiotics also govern the GM by selectively increasing the members of certain species while decreasing the colonization by members of other species. How a particular antibiotic asserts its influence depends on its type, dosage, exposure time, and mode of action (Rinninella et al. 2019).

## Gut microbiota and neurodegenerative disorders

With the advancement in GM research, there are several evidence suggesting the involvement of microbiota and its metabolites of the gut in regulating the development of neurodegenerative disorders like AD and PD (Chen et al. 2021b). In 1906, Alois Alzheimer described AD using its two prominent hallmarks namely amyloid-β plaques and accumulation of hyperphosphorylated tau protein (Schachter and Davis 2000). The common symptoms include memory decline in a progressive manner, personality and mood disorders, and sleep abnormalities. It is reported to be the most widespread neurodegenerative disorder with two types familial and sporadic (> 65 years of age) (Zhang et al. 2022). According to clinical research, GM has a role in the early pathophysiology of AD in humans. The changes in intestinal flora were found to be like those in the starting level of AD in the mild cognitive dysfunction stage, meaning that the diversity of fecal flora was much lower than in healthy individuals. This suggests that dysbacteriosis might be beneficial in the diagnosis of AD at the initial stages. A growing body of research has shown that AD and the inflammation caused by GM are closely related. The dysbiosis of GM in AD patients may bring about a decline in bacteria such as *Shigella* and *E. coli*, which are considered to be pro-inflammatory, and a surge in bacteria considered to be anti-inflammatory (Chen et al. 2021b). When the GM of AD patients and healthy participants were investigated, the AD patients displayed fewer populations of *Firmicutes, Proteobacteria, and Actinobacteria*, and an increased abundance of *Bacteroidetes.*

After AD, another prominent neurodegenerative disorder is PD, first described by James Parkinson as a progressive chronic disorder affecting the middle and elderly population (Zhu et al. 2022). Patients suffering from PD experience dysregulation of motor abilities including akinesia (absence of impulsive movement), difficulty in balance, rigidity, and frequent tremors. These motor dysregulations are attributed to a reduced dopamine level since PD majorly affects the dopaminergic neurons. The deposition of α-synuclein is the hallmark of this disease that eventually causes the death of neuronal cells. Alterations in the gut microbiota have started to gain substantial importance since PD has now been recognized as a multi-systemic disease with non-motor indications like constipation and paralysis of the stomach (gastroparesis) which develop before the motor abnormalities (Romano et al. 2021). Studies conducted earlier observed discrepancies amongst GM samples from patients with PD which can be due to differences in dietary habits, geographical location, and study design and method involved (Menozzi and Schapira 2024). Two SCFA-producing bacteria *Lachnospiraceae* and *Faecalibacterium* are observed to decline in patients with PD while *Lactobacillus* and *Bifidobacterium* are found to be enhanced (Romano et al. 2021).

## Conventional dietary restrictions and their effects on cognitive functions

Age-related pathologies have become frequent, and research has shifted towards preventing or reverting to age-related deterioration. A periodic reduction in calorie intake without causing malnutrition, termed Caloric restriction (CR) can help enhance longevity (Madeo et al., 2019). Studies have shown that CR improves brain health by enhancing cognitive function and reducing the detrimental effects of degenerative disorders (Halagappa et al. 2007).

Dietary restriction can induce neurogenesis in the dentate gyrus of the hippocampus, decrease neuronal cell death, and increase the expression of brain-derived neurotrophic factor (BDNF) (Lee et al. 2000). CR is also shown to attenuate the effects of neurochemical and behavioral deficits in rhesus monkey models of Parkinson’s disease potentially through the upregulation of glial cell line-derived neurotrophic factor (GDNF) and BDNF and therefore stimulating neuroprotective signal transduction pathways in dopaminergic neurons (Maswood et al. 2004). Mitochondria plays a vital role in mediating excitotoxicity, commonly observed in various neurodegenerative disorders. CR has been reported to induce mitochondrial adaptations and metabolic remodeling leading to protection against excitotoxicity (Amigo et al. 2017). Research conducted showed that nutrient starvation promotes autophagy in mammalian cells by decreasing acetyl CoA and therefore reducing the acetyltransferase activity of the E1A binding protein p300 (EP300) (Mariño et al. 2014). Further, another study was able to infer that CR could delay aging (Picca et al. 2013).

Another approach called intermittent fasting (IF) has been adopted for its influence on the gut-brain axis for ensuring healthy cognitive function. This approach unlike CR is focused on time-dependent calorie intake, alternating between hours of fasting and eating. IF exerts its beneficial impact by oscillating the relative population of gut microbiota in terms of timings of food intake. For instance, a time-restricted feeding pattern has been observed to have a rich diversity of gut microorganisms even without any prominent changes in nutrient consumption. However, there lacks concrete evidence to determine the positive impact of IF on a generalized population (Gudden et al. 2021) and such dietary restrictions are often accompanied by unwanted side effects like dizziness, headache, and fatigue (Shalabi et al. 2023). Although CR and IF promote health, their long-term implementation has been difficult. Hence alternative therapies that mimic their beneficial effects without a sustained decrease in food consumption are being investigated (Wahl et al. 2018).

## Caloric restriction mimetics (CRMs)

**Fig. 1:**
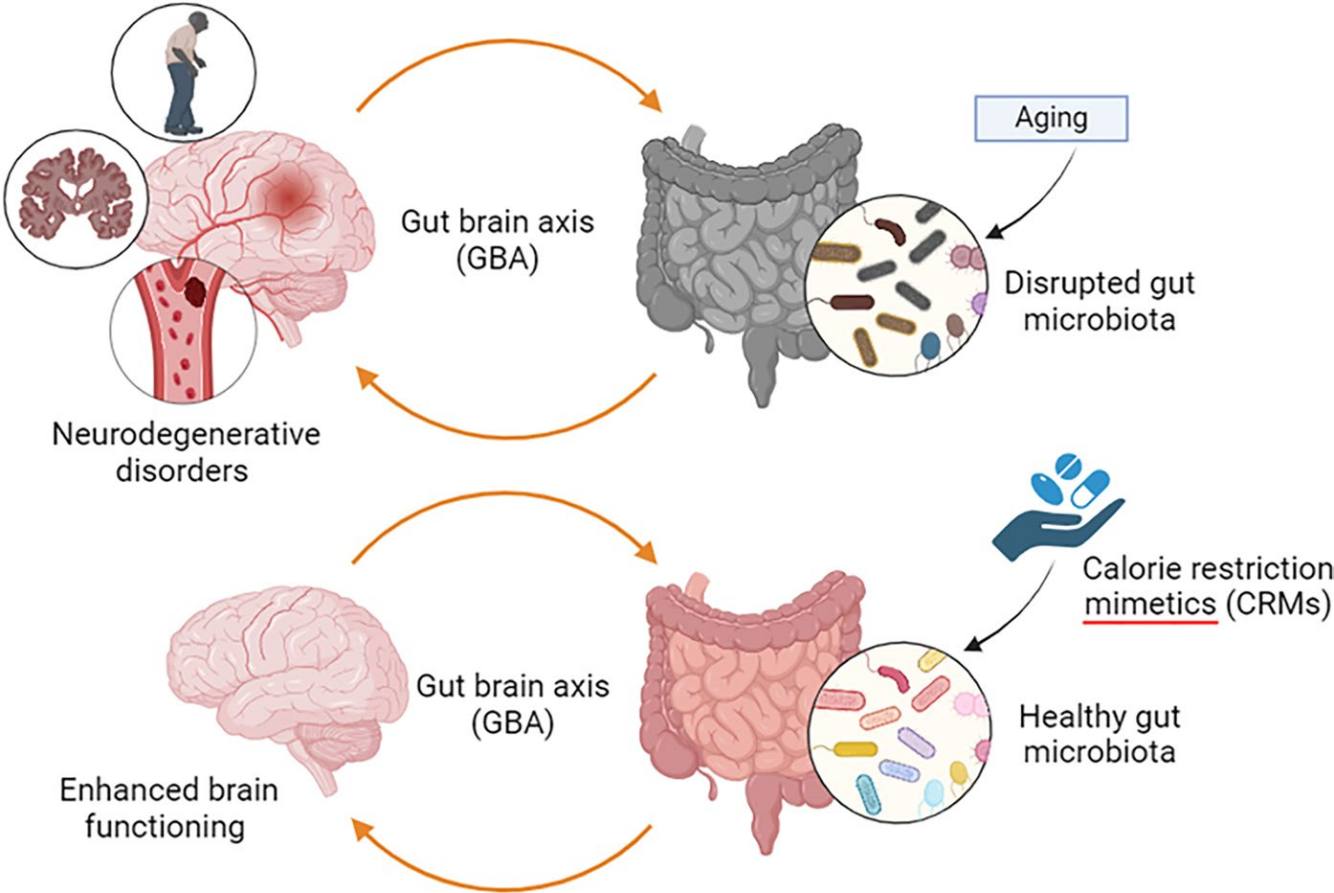
Aging is associated with disruption of normal gut microbiota. The gut-brain axis is a bidirectional axis that influences the functioning of the central nervous system; thus, disruption of gut microbiota can lead to neurodegenerative diseases. On the contrary calorie restriction mimetics (CRMs), through their influence on gut microbiota to secrete neurotransmitters and metabolites tend to enhance this bidirectional communication and lead to enhanced brain functioning

CRMs are pharmacological compounds that can replicate the beneficial activity of CR including physiological, metabolic, and hormonal effects. They do not significantly reduce food like CR. CRMs can initiate the pathways involved in stress response and increase resilience to stress. It can also mimic the action of CR on longevity and can reduce age-associated diseases. Lane et al. (1998) were the first to introduce the concept of CRMs through the study of the beneficial effects of 2-deoxy-D-glucose on biomarkers associated with aging in rodents (Lane et al. 1998). Based on the immediate action of several substances on mammalian cells, over ten medications have been classified as CRM in numerous studies to date. These are further classified as upstream-type CRMs, which inhibit the production of energy (Ingram and Roth 2011), while others are classified as downstream-type CRMs, which control or genetically modify intracellular signaling proteins (Ingram and Roth 2015). Moreover, CRMs promote healthy gut microbiota and thereby enhance brain functioning (Fig. 1).

### Mechanism of action of CRMs

**Fig. 2:**
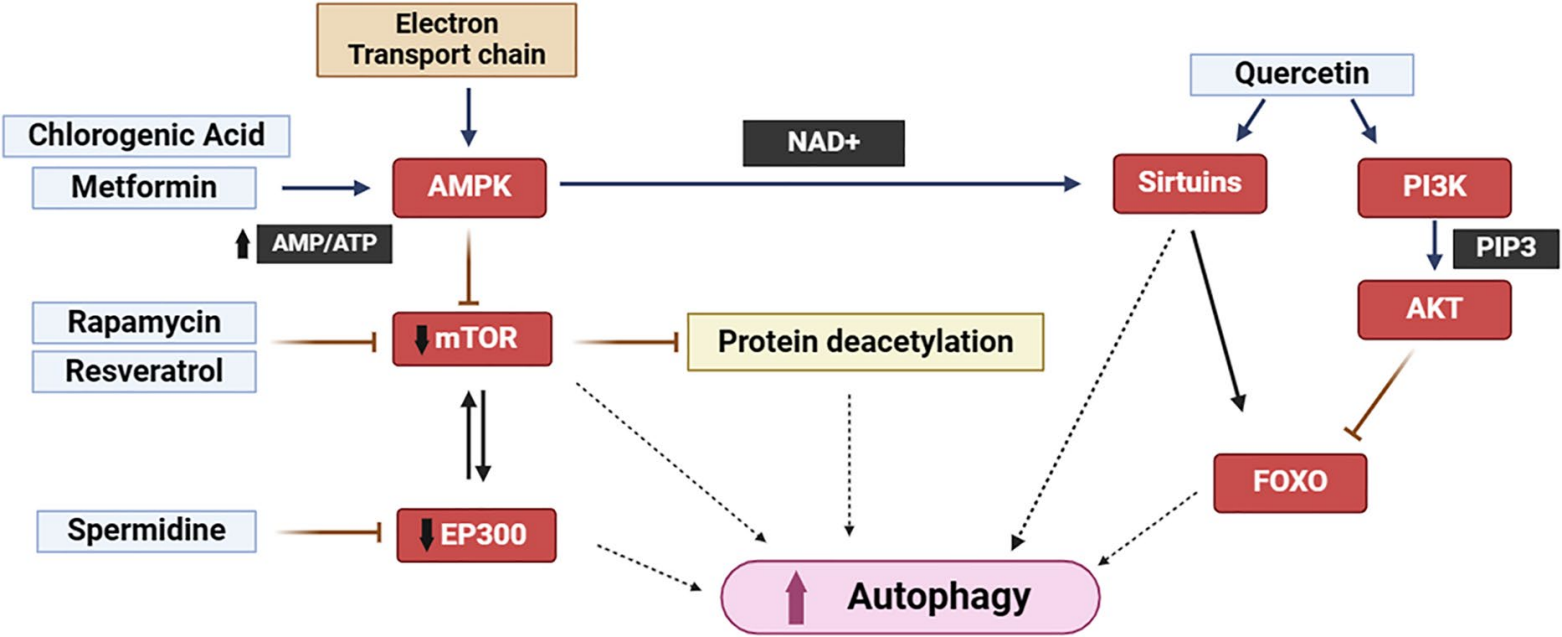
Various Calorie restriction mimetics (CRMs) and pathways they target. AMPK, Sirtuin, PI3K/Akt, and mTOR all are major pathways that are associated with autophagy. The action of different CRMs on these pathways helps to signal autophagy. AMPK and mTOR are sensor molecules, activation of AMPK and inhibition of mTOR both are responsible for the induction of autophagy. Sirtuin and PI3K also help regulate autophagy directly or indirectly through a signaling cascade

CRMs intend to provide similar benefits as traditional calorie restriction by acting on the molecular and cellular pathways that eventually lead to a decline in oxidative stress and initiate autophagy, the two major mechanisms affected as a consequence of aging and neurodegenerative disorders (Sharma and Singh 2023). Oxidative stress results as a consequence of the overproduction of free radicals such as hydrogen peroxide, superoxide radical, nitric oxide, hydroxyl radical, etc. (Atayik and Çakatay 2023)_._ Diffusible reactive oxygen species (ROS) such as hydrogen peroxide reacts with proteins sensitive to redox imbalance to modulate the metabolic pathways linked with redox changes (Homolak 2023b). Production of ROS in the gut leads to enhanced permeability of the intestinal barrier. Subsequently, this leaky gut results in inflammation and oxidative stress in ENS, which via the vagus nerve gets propagated to the CNS leading to neurodegeneration (Soni et al. 2024). A healthy human adult brain accounts for two percent of the total body mass, which is astonishing that even with such a low share the brain is accountable for the consumption of 20% oxygen and 20–25% of glucose (Steiner 2019). An extremely high metabolic requirement like this necessitates an efficient waste removal system so that the toxic by-products do not accumulate. However, the antioxidant defense and autophagy process decline during aging and are not able to cope with the increased level of ROS and toxic accumulation in the brain eventually leading to various neurovegetative disorders. The CRMs work by activating these pathways that have been suppressed because of aging, thus restoring the normal functioning of autophagy in the brain. The major routes through which CRMs function include AMPK, Sirtuin1, mTOR, and Keap1-Nrf2 pathways which have been highlighted in Fig. 2. Several CRMs working through the modulation of these pro-survival pathways is listed in Table 1.

**Table 1:** Various calorie restriction mimetics (CRMs) and their mode of action

CRM	Mode of action	Subjects	Dosage	Duration	References
Chlorogenic acid	Regulation of AMPK	Human adipocytes (in vitro) Male mice	10 μM 2 mmol/l CGA, IP	9 days 24 h	(Vasileva et al. 2020)
Fisetin	Inhibition of PI3K/Akt/mTOR Regulates SIRT1, Nrf2, NF-kB	human lung carcinoma A549 and H1792 cells Mice	5–20 μM (maximum inhibition showed by 20 μM) 20 mg/kg/day, IP	48 h 1 month	(Khan et al. 2012; Ahmad et al. 2021)
Spermidine	Positively modulates autophagy genes (ATG, BECLIN) and negatively regulates genes associated with inflammation (IL6)	Young and old male Wistar rats	10 mg/kg and	6 weeks	(Singh et al. 2021)
EGCG	Positive regulation of Nrf2 pathway Regulation of hippocalcin and intercellular calcium levels	Sprague–dawley rats	50 mg/kg	24 h	(Saha et al. 2020; Park et al. 2024)
Resveratrol	Activation of sirtuin (SIRT1), AMPK	Humans Humans	5 g single dose 1000 mg/day	24 h 28 days	(Gualdoni et al. 2014; Rege et al., 2014, 2019; Espinoza et al. 2017; Madeo et al.; Gabandé-Rodríguez et al. 2020)
Curcumin	Regulation of AMPK and sirtuin	Rats	200 mg, Oral	4 weeks	(Singh et al. 2023)
Hydroxycitric acid	Autophagy stimulation	*Saccharomyces cerevisiae*	5 mM	11 days, viability started declining after 6 days	(Baroni et al. 2020)
Metformin	Activation of AMPK through inhibition of mitochondrial respiration	BALB/c and CB17/icr-SCID mice	4 mM	7 weeks	(Madeo et al. , 2019; Gabandé-Rodríguez et al. 2020; Takahara et al. 2022)
2-Deoxyglucose	Inhibitor of glycolysis	Male wistar rats	25 mg/kg	12 weeks	(Kumar et al. 2020)
Rapamycin	Inhibitor of mTOR	Humans	2 mg/m^2^/day	Every 2 weeks for 18 weeks	(Madeo et al., 2019; Gabandé-Rodríguez et al. 2020; Mandrioli et al. 2023)
Gallic acid	Activation of AMPK pathway	HepG2 cells (in vitro)	50 μM	12 h	(Doan et al. 2015)
Kaempferol	Inhibitor of NF-κΒ pathway and SIRT1 activation	male wistar rats	200 mg/kg	8 weeks	(Alshehri et al. 2022)
Anacardic acid	Via EP300, induces IL-33	C57/B6 mice (both male and female)	10 µM	9 weeks	(Sharma and Singh 2023)
Acarbose	α-glucosidase	Male Wistar rats	30 mg/kg	6 weeks	(Smith et al. 2021; Arya et al. 2023)
Iodoacetate	Glycolysis inhibitor	Hippocampus cells from sprague–dawley rats	2, 20, 200 and 2000 nM	24 h	(Guo et al. 2001)
Adiponectin	Targets AMPK	Mice	1 mg/kg	6 and 3 h	(Kubota et al. 2007)
Quercetin	Regulator of PI3K/Akt, mTOR; Potent antioxidant; inhibitor of PIKδ kinase	Mice	20 mg/day, 25 mg/kg 500 mg/kg/day	2 weeks 2 days for 3 months 10 days	(Zhang et al. 2020; Ghafouri-Fard et al. 2021)
Garcinol	Action through EP300	Male sprague–dawley (SD) rats	20 mg/kg/day	3 days	(Kang et al. 2020)
Berberine	Works through sirtuin1	Mice rat	5 mg/kg/day 380 mg/kg/day	26 days 2 weeks	(Lee et al. 2006; Madeo et al., 2019; Sharma and Singh 2023)
Caffeic acid	Activator of AMPK and sirtuin	Mice	50 mg/kg/day	2 weeks	(Vasileva et al. 2020; Khan et al. 2023)

### Mammalian target of rapamycin (mTOR) pathway

mTOR is associated with mTORC1 and mTORC2 complex. The upregulation of the mTORC2 complex leads to neurodegenerative disorders through its potential to inactivate the Unc-like Kinase (ULK) complex, an initiator complex of autophagy (Mayor 2023). Under normal conditions, i.e. when nutrients are sufficient, mTOR being a serine/threonine kinase phosphorylates ULK1 and autophagy gene 13 (ATG13), members of the ULK complex, which leads to inhibition of autophagy. However, during stress or nutrient deprivation, mTOR is inactivated leading to the induction of autophagy. The self-degradative mechanism of autophagy is crucial for maintaining balanced energy sources at crucial developmental stages and in response to stress. Additionally, autophagy removes intracellular pathogens, misfolded or aggregated proteins, and damaged organelles such as the mitochondria, endoplasmic reticulum, and peroxisomes and plays a housekeeping role. Autophagy hence promotes cellular senescence (Glick et al. 2010). Rapamycin induces dephosphorylation of ULK1 and ATG13, mimicking the indirect inhibition of mTOR and initiation of autophagy (Liénard et al. 2024). Yet another noticeable regulator of mTOR is PI3K/Akt signaling. PI3K activation phosphorylates Akt protein which results in the activation of mTOR and consequently inhibition of autophagy (Kma and Baruah 2022).

Enhanced mTOR activity is also linked to insulin resistance, while calorie restriction and short-term rapamycin treatment enhanced insulin sensitivity and glucose uptake. The chronic inhibition of mTOR is shown to be associated with insulin resistance and glucose intolerance and may also lead to type 2 diabetes. These adverse metabolic effects put restrictions on the use of mTOR inhibitors as CRMs. Metformin can be used to reverse insulin resistance induced by mTOR inhibitors. The mode through which metformin acts, although partially understood, is by its inhibition of the mitochondrial respiratory chain (Kirpichnikov et al. 2002). This inhibition leads to a decline in cellular energy, which upregulates the AMP-activated protein kinase (AMPK) and furthers the inhibition of mTORC1 signaling in the liver (Stephenne et al. 2011).

### AMPK pathway

AMPK is a sensor molecule that senses variations in cellular energy, for instance, a change in the AMP and ATP ratio. It comprises 3 subunits: catalytic subunit α along with two regulatory subunits β and γ. AMPK gets activated when the cellular energy level decreases by phosphorylation of its threonine residue in the catalytic subunit. CRMs activate AMPK by leading to a decline in ATP production, thus increasing the AMP to ATP ratio. Activation of AMPK is essentially considered important because of its role in anti-aging and neuroprotection by regulation of autophagy and oxidative stress reduction. AMPK can directly or indirectly initiate autophagy by downregulation of the mTOR pathway, which inhibits autophagy when activated (Sharma and Singh 2023). AMPK directly activates ULK1, a crucial complex for autophagosome formation by phosphorylating it. It can also indirectly associate with mTORC1 inhibiting it from phosphorylating ULK1, thus initiating the autophagy process. Apart from this AMPK is involved in phosphorylation and activation of FOXO, a transcription factor. Phosphorylation of FOXO at six of its regulatory sites by AMPK leads to gene transcription which plays a role in stress regulation (Greer et al. 2009).

Resveratrol, a potent CRM can exert its effect indirectly through the activation of AMPK (Cantó et al. 2010). It was observed that resveratrol can improve the lifespan of mice through increased AMPK level (Baur et al. 2006). A 30-day study was conducted on obese humans to understand the effects of resveratrol (resVida^TM^) on their energy metabolism and metabolic profile. They found that supplementing resveratrol to humans resulted in beneficial metabolic changes, such as decreased blood pressure, hepatic lipid content, sleeping metabolic rate, and intrinsic mitochondrial function in skeletal muscle, improved intramyocellular lipid content, and increased peroxisome proliferator-activated receptor- γ coactivator 1α (PGC-1α) protein content in skeletal muscle (Timmers et al. 2011).

### Sirtuin1 (SIRT1) pathway

Sirtuin are deacetylases dependent on nicotinamide dinucleotide (NAD^+^) and are categorized into SIRT1 to SIRT7. They are found in different locations inside the cell, with SIRT1 majorly localized in the nucleus (Hassani et al. 2022). For SIRTs to be activated, the presence of NAD^+^ is a must, it thus acts as a sensor molecule responding to changes in cellular energy levels (Mayor 2023). SIRT1 asserts its role by deacetylation of lysine residues of proteins like Forkhead box subgroup O (FOXO), a transcription factor involved in the transcription of autophagy genes (Hassani et al. 2022). Research incorporating animal models has shown that through the deacetylation of its target proteins including transcription factors and transcriptional coregulatory proteins, SIRT1 affects a variety of biological processes, such as gene silencing, stress resistance, mitochondrial biogenesis, glucose and lipid metabolism, autophagy, cell survival, apoptosis, and inflammation (Michan and Sinclair 2007). SIRT1 can further activate AMPK through the deacetylation of a molecule located upstream of AMPK, liver kinase B1 (LKB1), situated upstream of AMPK (Price et al. 2012). CRMs can function as activators of protein (de)acetylases, particularly, SIRT1. It was found that resveratrol is the most potent activator of SIRT1, and it can exert direct effects on SIRT1 (Howitz et al. 2003).

### Nrf2-Keap1 pathway

**Fig. 3:**
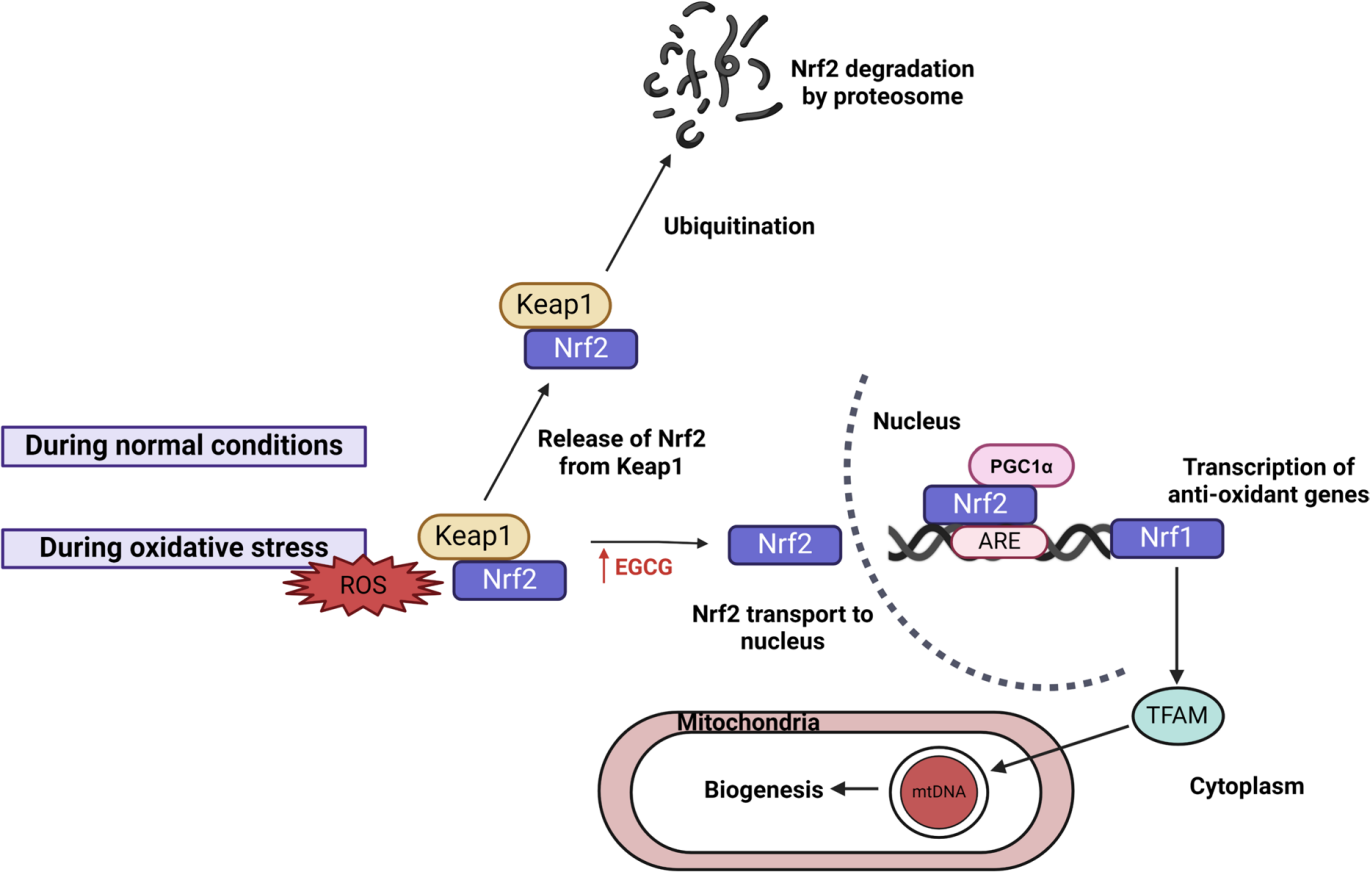
Nrf2-Keap1 pathway and the CRM-like ECEG leading to activation of transcription of antioxidant genes by Nrf2. Normally Nrf2 is sequestered by Keap1 in the cytoplasm leading to its degradation by proteasome. However, during oxidative stress, Nrf2 dissociates and proceeds to the nucleus leading to the transcription of genes involved in antioxidation. The association of Nrf2 with PGC-1α leads to the functioning of Nrf1 which is directly related to the process of biogenesis of mitochondria via TFAM (Transcription Factor A, Mitochondrial). EGCG is a calorie restriction mimetic that helps Nrf2 dissociate from Keap1 thus activating the antioxidation process

The nuclear factor erythroid 2–related factor 2- Kelch-like ECH-associated protein 1 (Nrf2-Keap1) pathway is a crucial pathway for managing oxidative stress (Ji et al. 2015). Nrf2 is a very essential transcription factor associated with autophagy genes. During normal conditions, Nrf2 is present in the cytoplasm bound to Keap1 which prevents its activation and mediates its degradation. However, under conditions of stress, for instance, an increase in ROS, Nrf2 can disassociate from Keap1 and transport to the nucleus. Within the nucleus, it binds to autophagy response elements (ARE) located in the promoter region of the transcriptional unit of antioxidants and facilitates their transcription (Fig. 3).

CRMs can be used to target Keap1, leading to its disassociation from Nrf2. This can be achieved by modulating the cysteine residues in Keap1 (Sharma and Singh 2023). Research conducted by Ji et al 2015 showed that Quercetin, a CRM led to the activation of Nrf2 and induced expression of antioxidant enzymes. Another study demonstrated the ability of ECEG as a CRM by upregulating Nrf2 and antioxidant activity (Ma et al. 2021). While Nrf2 activation helps protect from oxidative damage, another molecule PGC-1α in association with Nrf2 helps regulate the biogenesis of mitochondria. PGC-1α in cooperation with Nrf2 leads to activation of Nrf1. Nrf1 further activates the mitochondrial transcription factor A (TFAM), the key regulator of mitochondrial replication.

Apart from the major pathways, CRMs activate autophagy by increasing the deacetylation of cellular proteins, depleting AcCoA, inhibiting acetyltransferases, and/or facilitating deacetylases. Starvation depletes AcCoA and in turn, inhibits acetyltransferase EP300. It also increases NAD^+^/NADH and AMPK activation leading to activation of SIRT1 deacetylase. This causes the deacetylation of proteins in turn activating the autophagic cascade (Morselli et al. 2011). Pietrocola et al. (2018) classified CRMs into three major classes based on their mode of action. The first class of CRMs directly inhibits acetyltransferases and primary autophagy repressor histone acetyltransferase EP300/ep300. Secondly, CRMs can indirectly affect the enzymatic activity of acetyltransferases by inhibiting the biosynthesis of acetyl coenzyme A (AcCoA) which would deplete the sole donor acetyl moieties. Thirdly, CRMs can function as activators of protein (de)acetylases, particularly, SIRT1 (sirtuin 1). For instance, Aspirin and salicylate (an aspirin metabolite) can induce autophagy by inhibiting the EP300 enzyme activity (Pietrocola et al. 2018). As a competitive inhibitor of ATP citrate lyase (ACLY), the CRM hydroxy citric acid (HCA) depletes acetyl-CoA pools, by cleaving citrate to oxaloacetate and AcCoA and hence promoting autophagy through the suppression of protein acetylation (Hoffmann et al. 1980).

## CRMs and their effect on neurodegenerative disorder

CRMs activate several pro-survival pathways and provide neuroprotection against age-associated neurodegenerative disorders through the activation of the autophagy process. In addition, CRMs also activate antioxidant mechanisms to maintain redox homeostasis in the brain.

### Alzheimer's disease

One age-related neurodegenerative disorder that has been steadily rising in recent years is Alzheimer's disease (AD). Around 55 million individuals worldwide have dementia, according to a WHO report from 2023. The occurrence of neurofibrillary tangles, abnormal levels of Tau protein accumulation, and amyloid-beta (Aβ) plaques are defining features of AD (Wilson et al. 2023). It has also been shown that dysregulation of mucus production in the gut, which forms the primary defense against infections, is impaired in AD. This impairment further affects the permeability of the intestinal lining leading to the transfer of toxic accumulates and ROS to the brain via the vagus nerve (Homolak et al. 2023). These abnormalities cause massive neuronal degeneration, which ultimately results in the shrinkage of multiple regions of the brain and connections involved in various cognitive functions (Ahmad et al. 2023). Although there are treatments available that merely aid with symptoms, there is still no known cure for AD (Peng et al. 2023). Evidence from animal and in vivo studies has demonstrated that calorie restriction shows a beneficial effect in resorting to the hallmarks of AD (Wu et al. 2008). SIRT1-mediated deacetylation of FoxO in response to CR has been shown to increase the α-secretase activity, decreasing Aβ plaque deposition by inhibiting the Rho-associated protein kinase-1 (ROCK1) (Qin et al. 2008). CR represses the activity of cyclin-dependent kinase 5 (Cdk5). Cdk5 is activated when the level of p25 increases which leads to the generation of hyperphosphorylated tau (Seo et al. 2017). Also, this CR suppresses the activity of mTOR by activating the AMPK. It has been suggested that AMPK is a potential therapeutic target for disorders including obesity, Type 2 diabetes, and neurodegeneration. Studies have demonstrated that AMPK engages in controlling both tau phosphorylation and Aβ production, and AMPK signaling plays a significant role in AD pathology (Yang et al. 2020a). Some CRM drugs are showing a potentially effective role in curing the symptoms of AD (Madeo et al., 2019). Metformin has been shown to improve mitochondrial morphology in human neural stem cells (hNSCs) by activating AMPK-acting neuroprotective agents against the Aβ (Chiang et al. 2016). In P301S mice, metformin has shown its effect in reducing tau phosphorylation by inducing protein phosphatase 2A (PP2A) expression via the AMPK/mTOR pathway (Barini et al. 2016). So, there is much evidence suggesting a therapeutic role of metformin for neurodegenerative disorders. Resveratrol mimics the CR by exhibiting neuroprotection through activation of the STIR2 (Morselli et al. 2010).

### Parkinson’s disease

According to a 2019 WHO survey, 85 million individuals worldwide suffer from Parkinson's disease (PD), which renders it one of the most prevalent disorders. An overabundance of misfolded α-synuclein proteins and the deterioration of dopaminergic neurons in the substantia nigra are distinctive hallmarks of PD. This results in an overall reduction in the neurotransmitter dopamine, which in turn impacts motor function and produces symptoms, the primary ones being bradykinesia, ataxia, tremor, and stiffness (Antony et al. 2013). Moreover, dopamine reduction is also attributed to the dysregulation of GM, triggering an increase in ROS level and pro-inflammatory molecules, which consequently leads to α-synuclein deposition prorogation from the gut to the brain (Soni et al. 2024). CR has shown its effects in maintaining the symptoms of PD in animal models i.e. elevated dopaminergic neuron (DA) survival in the substantia nigra, higher neuronal survival, and brain-derived neurotrophic factor (BNDF) leading to improvement in the motor functions (Maswood et al. 2004). By stimulating the AMPK–autophagy signaling pathway, metformin (CRM drug) lowers the risk of PD and promotes neurogenesis and the establishment of spatial memory (Lu et al. 2020).

## Interaction between calorie restriction mimetics and gut microbiota

### CRMs on improved gut barrier function

**Fig. 4:**
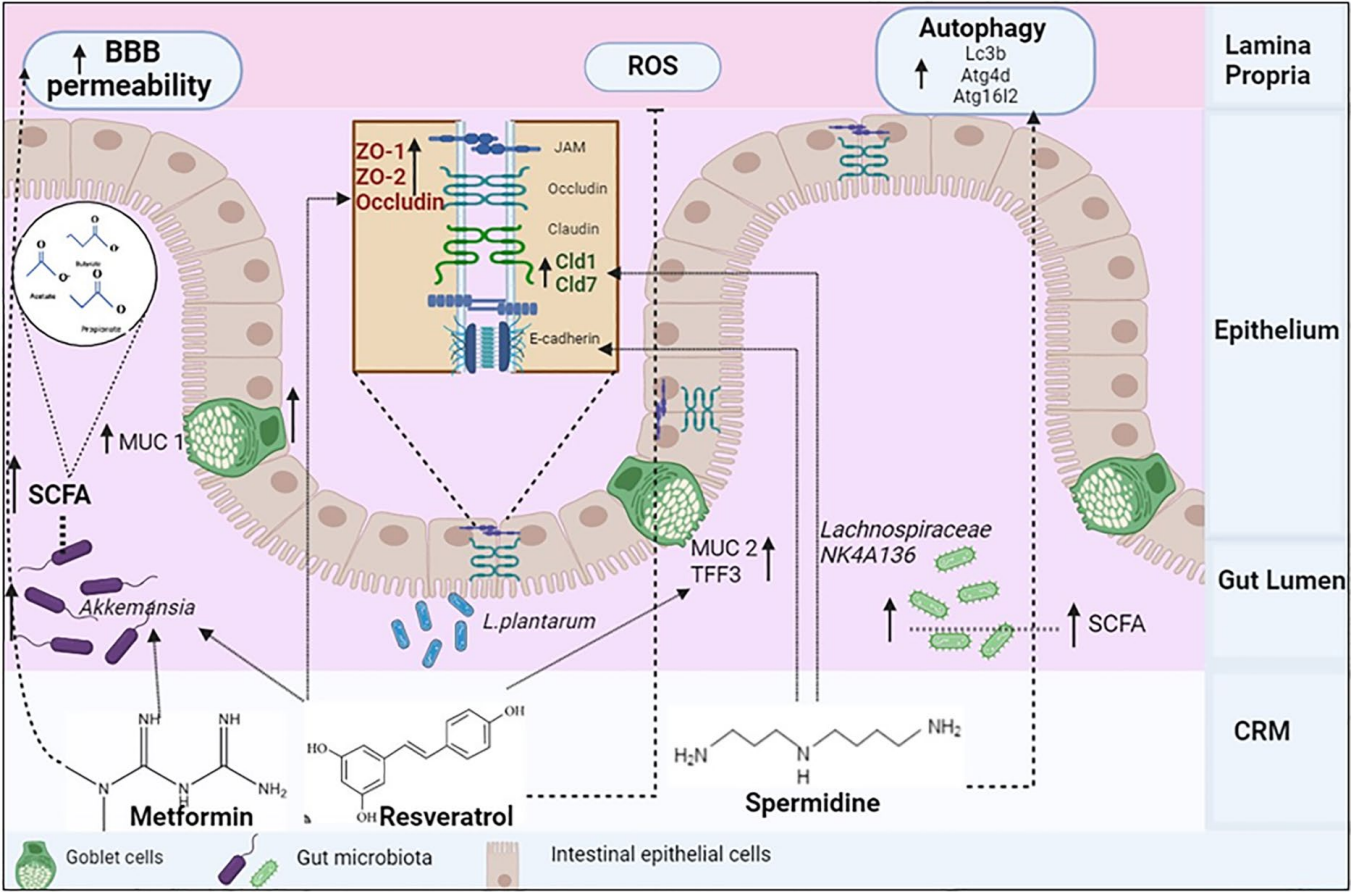
Effect of various calorie restriction mimetics (CRMs) on improved gut barrier function. CRMs like metformin, resveratrol, and spermidine stimulate the gut microbiota to secrete metabolites like SCFA, neurotransmitters that have potential benefits in enhancing blood–brain barrier permeability, managing oxidative stress, and induction of autophagy

The human intestinal tract consists of various gut flora such as *Bacillota, Bacteroidota, Actinomycetota*, *Pseudomonadota,* and *Verrucomicrobia* which maintain the intestinal barrier (Hou et al. 2022). The intestinal barrier consists of an outer layer of complex sugar compounds in mucous form and the inner layer consists of epithelial cells (Kelly et al. 2015). Dietary habits, living environment, and age are some of the variable factors that affect gut microbial activity (Moran-Ramos et al. 2020), which induces the thinning of the outer layer, where components of bacteria come in contact with dendritic cells leading to the release of cytokines, ultimately resulting in loosening of tight junction (Kelly et al. 2015). It is intriguing to see that gut flora gradually alters as we age (Madeo et al., 2019). A variety of studies have demonstrated that CRM compounds have a beneficial effect on the regulation of the aging process by utilizing intestinal bacteria and their metabolites (Shintani et al. 2023). Dimethylbiguanide, or metformin, is a guanidine molecule that is one of the identified CRM potential drugs (Bailey 2017). With its widespread usage in the treatment of type 2 diabetes, metformin, and D-allulose have been found to improve gut flora's glucose metabolism via activating AMPK (Shintani et al. 2017). Metformin has demonstrated enhanced benefits on cognitive performance and has been proven to reduce leaky gut by upregulating the production of the tight junction protein mucin1. Additionally, older mice treated with metformin showed an increase in beneficial bacteria (Ahmadi et al. 2020). These tight junction proteins expressed at the cell–cell junction help maintain the intestinal barrier (Chelakkot et al. 2018). Moreover, it also has shown its effect in improving the BBB barrier in ischemic stroke (Liu et al. 2014). One prospective CRM drug, resveratrol, has been shown to strengthen gut permeability and cell junction integrity by upregulating the ZO-1, ZO-2, occludin proteins (Wang et al. 2016) and also increasing the expression of mucin2 (MUC2) and trefoil factor 3 (TFF3) which are involved in regulating the mucosal integrity (Likhitwitayawuid 2021). Resveratrol, on the other hand, can mitigate damage caused by ROS by elevating the production of tight junction proteins, which are dependent on the Nrf2 signaling pathway controlled by PI3K/Akt (Song et al. 2022). A study conducted by (Zhu et al. 2023) revealed that the metformin elevated *Akkermansia muciniphila* (*A. muciniphila*) enhanced cognitive performance in older mice by lowering inflammation in the hippocampal region. In another study conducted on *C. elegans*, metformin retards the aging process by interfering with *E. coli’s* ability to metabolize folate (Cabreiro et al. 2013) and D-allulose has been shown to extend the lifespan (Shintani et al. 2017). Consequently, improper glucose management could accelerate the aging process. Similarly, acarbose is also involved in regulating glucose metabolism through the MAPK pathway (Zhang et al. 2013). Polyamines such as spermidine, homospermidine, and putrescine participate in maintaining gut permeability by inducing the cell–cell adhesion protein E-cadherin. Therefore, these CRM compounds aid in the preservation of the intestinal barrier (Fig. 4).

A study investigated three plant-derived flavonoids, quercetin, catechin, and puerarin, for their effects on gut microbiota in vitro. They found that quercetin stimulated *Firmicutes, Proteobacteria*, and *Actinobacteria*, whereas puerarin enhanced *Fusobacteria* and *Proteobacteria*, increasing the diversity and abundance of microbial communities (Huang et al. 2016). In a study that stimulated anaerobic human fecal fermentation in vitro, it was shown that green tea increased the populations of *Lactobacilli spp*. and *Bifidobacteria spp* (Rha et al. 2019).

## Effect of CRMs on metabolite production by gut microbiome

**Fig. 5:**
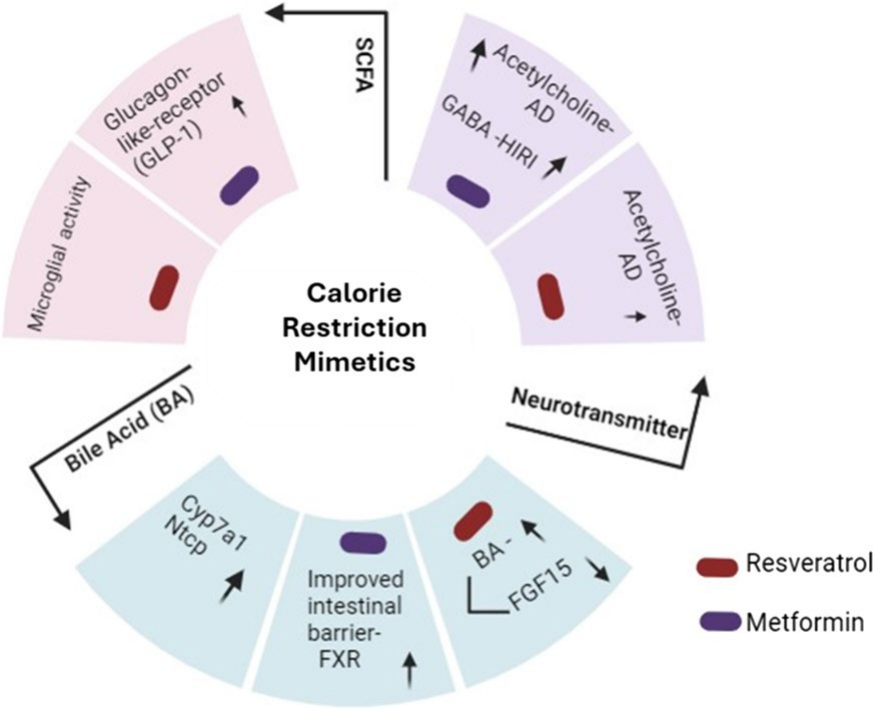
Calorie restriction mimetics resveratrol and metformin effect on the release of neurotransmitters, bile acid (BA), and short-chain fatty acid (SCFA). Resveratrol and metformin through their action as CRM regulate the gut microbiota into release of these metabolites that help enhance the functioning of the brain

Metabolites for instance fatty acids, trimethylamine-*N*-oxide, tryptophan, and polyphenolic compounds are metabolized by the GM from the food. It also helps produce and metabolize bacterial metabolites such as lipopolysaccharide. These metabolites generated by the GM can modulate the redox levels and influence the physiological processes of the body (Zhao et al. 2023). Thus, a change in GM composition is likely to affect metabolite production and lead to an imbalance in redox homeostasis. Experimental evidence has suggested that in epithelial cells ROS is generated as a response to *Lactobacilli* which leads to a signaling cascade inactivating regulatory proteins through redox reactions (Jones and Neish 2017). CRMs have been shown to improve the composition of the gut microbiome which leads to changes in metabolite production such as short-chain fatty acid (SCFA), bile acid, and neurotransmitters. These metabolites are crucial for maintaining brain health through several mechanisms (Fig. 5).

### Short-chain fatty acid (SCFA)

A microbial compound SCFA is obtained from gut bacteria when complex carbohydrates undergo saccharolytic fermentation of SCFA. SCFAs have a variety of local actions that promote gut health, including mucus synthesis, inflammation prevention, and intestinal barrier integrity preservation by upregulating the genes encoding claudin-1, zonula occludens-1 proteins, which helps in maintaining the gut barrier (Fusco et al. 2023). The research conducted by Mirzaei et al. 2021 showed that SCFA inhibits the histone deacetylase (HDAC), potentially affecting several neuropsychiatric conditions. Although it is well-established that SCFA metabolites support GM. Remarkably, current research points out that CRM drugs can use intestine bacterial metabolites to enhance the gut condition. A study showed that metformin increases the number of bacteria such as *Akkemansia,* and *Phascolarctobacterium* that produce SCFAs, which in turn stimulates the release of gut hormones like glucagon-like peptide-1 (GLP-1) thereby maintaining the intestinal barrier and promoting healthy aging and longevity. Resveratrol is one of the neuroprotective agents that has shown its effect by elevating the levels of SCFA-producing bacteria, which are involved in regulating microglial activity (Wenzel et al. 2020).

### Bile acids

Water-soluble steroidal molecules known as bile acids are released into the gastrointestinal system and can be converted into secondary bile acids by intestinal microorganisms (Cai et al. 2022). Bile acids activate the farnesoid X receptor (FXR) in the enterocytes which aids in the production of fibroblast growth factors (FGF). FGF19 is produced in the ileum because of the FXR being expressed in response to secondary bile acids. FGF19 can penetrate the BBB, enter the systemic circulation, and activate the hypothalamic arcuate nucleus (Hsuchou et al. 2013, p. 19). Metformin improved the integrity of the intestinal barrier by activating the FXR signaling in intestinal epithelial cells through *Lactobacillus* (Yang et al. 2022). Another FGF15 which acts as an FXR signal in the brain involved in regulating glucose metabolism (Liu et al. 2018). However, a study shows that resveratrol-induced bile acid synthesis by downregulating the enterohepatic FXR-FGF15 axis and increasing the amount of *Bacteroides, Lactobacillus*, and *Akkermansia* in mice (Chen et al. 2016). In the study, CRM alleviated the expression of Ntcp and Cyp7a1 gene which are involved in regulating the synthesis of BA (Gregor et al. 2021).

### Neurotransmitters

Current research indicates that the gut microbiota also produces several neurotransmitters, including acetylcholine (ACh), glutamate, gamma-aminobutyric acid (GABA), and serotonin (Strandwitz 2018). Some of the neurotransmitter precursors such as tyrosine and tryptophan travel to reach BBB and undergo a few intermediary processes before becoming a functional neurotransmitter. ACh is a cholinergic neurotransmitter that is produced by multiple bacteria i.e., *Bacillus acetylcholine, Staphylococcus aureus, Bacillus subtilis, Lactobacillus plantarum*, and *Escherichia coli.* In a study (Silamiķele et al. 2021), metformin treatment results in an increase in *Lactobacillus plantarum*, which is consistent with a recent study evaluating the impact of metformin on the GM connected to short-term obesity caused by a fat-rich diet (Ji et al. 2019). As we know this strain can make ACh, which is reduced to a very low level in AD patients (Ju and Tam 2022), and upregulation of ACh increased *Lactobacillus plantarum* which is one of the contemporary therapies (Athari Nik Azm et al. 2018). It has been observed that resveratrol possesses antioxidant capability, decreases inflammation, and declines cellular aging (Sawda et al. 2017). Considering these advantages, resveratrol with vitamin A showed an increased level of ACh in synapses to mediate anti-dementia medication for AD patients and also showed neuroprotection by regulating BDNF (Foudah et al. 2023). It was discovered that these gut bacteria *Parabacteroides, Bacteroides thetaiotaomicron, Bifidobacterium, Bacteroides unifomis,* and *Eubacterium* can produce the GABA, inhibitory neurotransmitter (Chen et al. 2021a). Through the use of 16S rRNA and metagenomic sequencing, it was demonstrated that metformin therapy elevated GABA levels by altering the composition of gut bacteria in cases of hepatic ischemia/reperfusion injury (Wang et al. 2019). Metformin has shown its effect in normalizing the tryptophan metabolism, and thereby serotonin level which is involved in regulating neural activity (Roth et al. 2021).

## CRMs and the gut-brain axis (GBA)

**Fig. 6:**
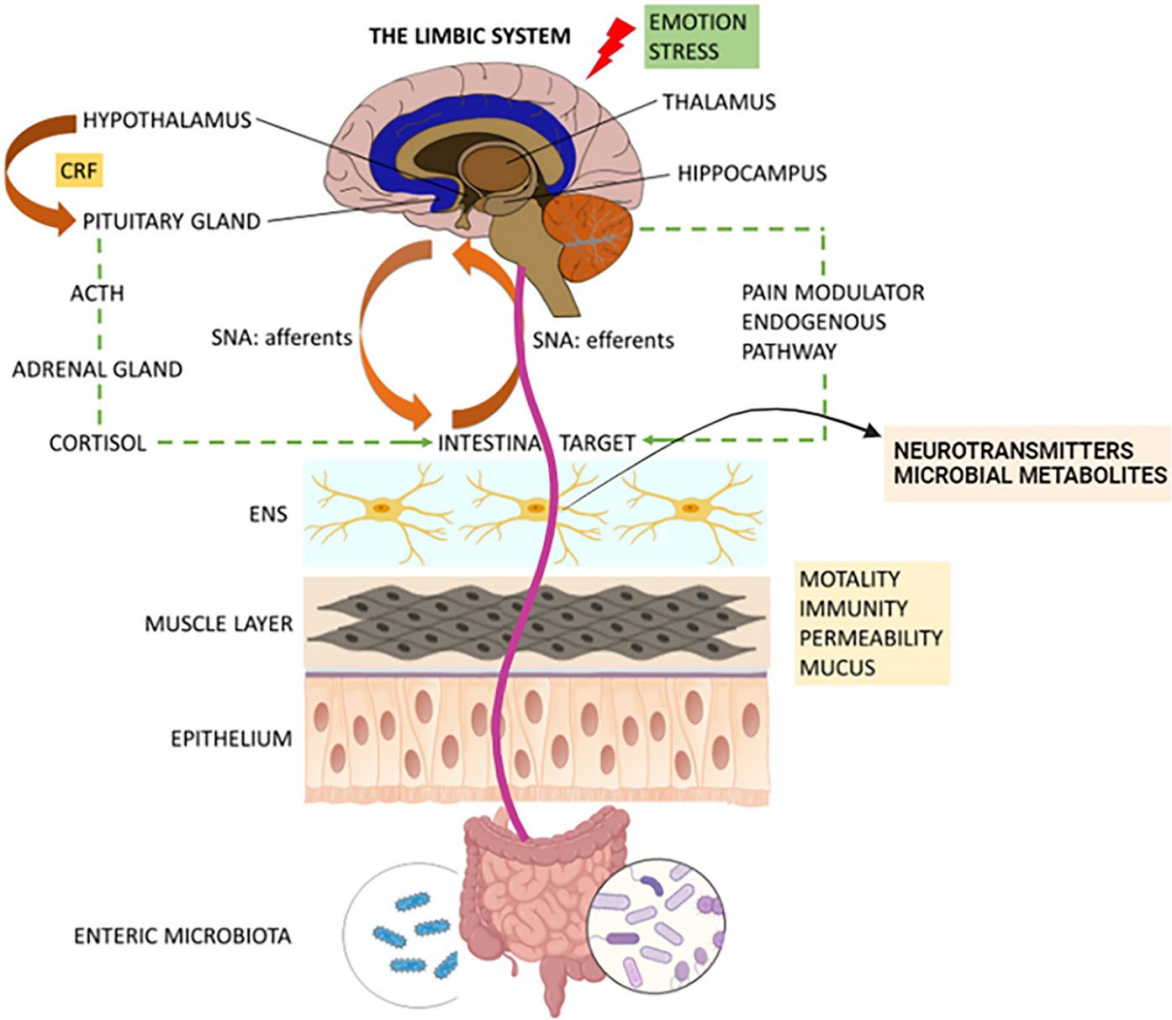
Gut brain axis (GBA). The GBA is a bidirectional axis that establishes communication between the gut microbiota and the workings of the brain

The transmission of signals from the gastrointestinal motor and sensory components to the central nervous system (CNS) forms a crucial aspect of the gastrointestinal-brain axis. Conversely, the response from the CNS back to the intestine can be defined as the GBA (Jones et al. 2006) (Fig. 6)**.**

Both clinical and experimental evidence indicate that the enteric microbiota plays a significant role in the GBA, not only by associating with the enteric nervous system (ENS) and intestinal cells but also on a local level. The colonization of microbiota and gut metabolites is impacted by nutrition, resulting in potential effects on brain development and function via neural, immunological, and endocrine pathways (Deverman and Patterson 2009). The GBA, serving as the central entity, encompasses the cerebral cortex, the limbic system, the hypothalamic-pituitary axis, and the brain system, all interconnected. The limbic system receives input from various brain regions, including the hippocampus, which plays a crucial role in a multitude of behaviors (Perlman 2002).

Over the past several years, there has been an increase in the number of animal-based experiments designed to investigate the role of the microbiota in regulating GBA. Various technological approaches have also been employed, such as the use of probiotics, antibiotics, infection research, and germ-free (GF) animals. Microbial inhabitation of the gut is essential for the development and maturity of the ENS and CNS, according to research in GF animals (Barbara et al. 2005). The absence of microbial colonization is linked to changes in gut sensory-motor functions, including slowed down gastric and intestinal transit, decreased migratory motor complex cyclic recurrence and distal propagation (Husebye et al. 2001), and enlarged cecal size. It is also linked to altered neurotransmitter expression and turnover in both nervous systems (Heijtz et al. 2011). Enzymes involved in the production and transport of neuromuscular substrates have lower gene expression in neuromuscular disorders, as well as in the case of muscular contractile proteins. According to the study conducted on GF animals, microbiota affects stress reactivity, anxiety-like behavior (Clarke et al. 2013), and the set point for HPA activity. The animals under study showed decreased anxiety and increased response with elevated levels of ACTH and cortisol. The gut undergoes a process of microbial colonization, resulting in the restoration of the axis in a manner that is dependent on age. The exaggerated stress response, observed after colonization by microbes, is reversible only in very young mice. This observation supports the notion that there exists a crucial interval at which neural plasticity is susceptible to microbiota (Sudo et al. 2004). In addition, in genetically modified animals, there has also been documentation of impaired cognitive function, potentially attributed to a modified expression of BDNF. It is a key determinant in memory and is predominantly found in the hippocampus and cerebral cortex. This molecule governs various aspects of brain function and cognitive processes, as well as muscle recovery, regeneration, and differentiation (Al-Qudah et al. 2014). The microbiota's presence ultimately leads to the modulation of the serotoninergic system as evidenced by the surge in serotonin turnover and dysregulation associated metabolite levels within the limbic system of GF animals. The influence of gut microbiota on GBA is supported by the study, which can manipulate gut microbes using probiotics and antibiotics. These investigations additionally validate the fact that the microbiota has an impact on anxiety and the HPA system through its influence on brain neurochemistry.

The constitution and total biomass of the enteric microbiota are subject to modulation by various psychological stressors, irrespective of their duration. The short stressors affect the gut microbiota and can change the profile of the community and relative proportions. The potential impact of these effects can be regulated by the interconnected neuroendocrine output efferent systems, specifically the autonomic nervous system (ANS) and HPA. This regulation can occur either through direct communication between the host-enteric microbiota or through alterations in the intestinal environment. The efferent neural pathways, which are connected to the endogenous pathways responsible for pain modulation, are collectively referred to as the "emotional motor system" (Rhee et al. 2009). Additionally, the brain is a key determinant in controlling gut functions like motility, mucus, acid, and bicarbonate secretion, maintaining intestinal fluid, and mucosal immune response. These processes are all critical for maintaining the mucus layer and biofilm, which are layers of mucus formed by individual bacterial groups growing in a variety of distinct microhabitats and metabolic niches connected to the mucosa (Macfarlane and Dillon 2007). The disruption of the typical mucosal environment caused by a dysregulation of GBA might therefore have an impact on the gut flora. Acoustic stress exerts an influence on the postprandial motility of the gastric and intestinal regions in canines, resulting in a prolongation of the recuperation time required for the restoration of the migrating motor complex pattern and instigating a temporary deceleration in the process of gastric emptying (Gué et al. 1989). Mental strain also amplifies the occurrence of spike-burst activity in the cecocolonic region by the central release of corticotropin-releasing factor (CRF) (Gue et al. 1991). The intestinal barrier and the BBB are the main barriers to GBA signaling. These barriers possess a dynamic nature, and various elements such as the gut microbiota, inflammatory signals, and stress possess the ability to regulate their permeability. In a state of good health, both barriers exhibit a high level of integrity, effectively impeding the transmission of immune signals from the microbiome to the brain.

## Experimental and clinical evidence of CRMs

### Effect on body weight and composition

A natural CRM obtained from turmeric called curcumin has been studied for its benefit on the reduction of weight. Data from 8 clinical trials conducted on individuals with an age of more than 18 years and an average population body mass index (BMI) of 25 kg/m^2^ suggested a declining effect on overweight and obesity (Hariri and Haghighatdoost 2018). It was also noted that curcumin administration with extended intervention led to reductions in visceral fat and total body fat (Chuengsamarn et al. 2014). Other CRMs like Epicatechin and EGCG, collectively known as catechins were also found to have the potential to reduce BMI and Body weight even at low doses as suggested by meta-analyses of data from dispensable clinical trials (Phung et al. 2010; Kapoor et al. 2017).

### Inflammatory response

A well-known CRM D-glucosamine which works as an inhibitor of glycolysis by blocking the hexokinase-1 enzyme of the glycolytic pathway is known for its role in anti-inflammatory response. A randomized control trial performed for a duration of 4 weeks administering 1.5 g a day of the drug combined with chondroitin sulfate (1.2 g a day) found the levels of C-reactive protein (CRP) to be reduced (Navarro et al. 2015). This observation was also supported by another study on glucosamine suppressive response on pro-inflammatory mediators for instance IL6 in human and animal models (Largo et al. 2003). Yet another CRM that has been majorly studied for its anti-inflammatory action is curcumin. Curcumin administration showed a decline in biomarkers of inflammation such as IL6, CRP, and TNFα and a subsequent inclination of markers of anti-inflammatory action such as IL10, as evidenced by the data collected and meta-analyzed from trials (Ferguson et al. 2021). The effect of resveratrol was studied on 119 patients and was found to have a modulating effect on neuroinflammation and a decline in Aβ42 and Aβ40 (Moussa et al. 2017).

### Cognitive performance

Polyamines, for instance, spermidine have been widely recognized as CRMs. A pilot study was conducted on elderly people to test the effects of polyamines like spermidine, putrescine, and spermine (1.2, 0.2 and 0.6 mg each day respectively) for a period of 3 months. It showed improved results for memory performance (Wirth et al. 2018). In another study, spermidine was administered with diet for a duration of 3 months (3.3 mg) to elderly people with mild dementia residing in nursing homes and observed an enhancement in cognitive abilities (Pekar et al. 2021). Niacin, which serves as a dietary alternative for NAD^+^ precursor has been found to have positive effects on cognitive performance. A study done administering 0.25 g of niacin each day for a duration of 1.5 months in patients suffering from PD, showed enhanced cognitive and sleep cycles as well as their motor abilities were also found to be improved (Wakade et al. 2015). Another study conducted on 92 older individuals, two groups, double-blinded, multicentric, and longitudinal to check the effectiveness of spermidine found to have an improved cognitive performance (Pekar et al. 2021).

## Neurotransmitters

With aging, there is a substantial change in the levels of monoaminergic neurotransmitters such as dopamine, 5-hydroxytryptamine, and norepinephrine. It has been found that the level of dopamine is reduced in the hippocampal regions of the aged rat brain. Similarly, 5-hydroxytryptamine and norepinephrine are also found to be downregulated in the hippocampus and striatum of old rats (Portero-Tresserra et al. 2020). Studies have shown that CRMs like rapamycin can restore dopamine levels in brain regions (Prvulovic et al. 2022). Another prominent CRM, quercetin (10 and 20 mg/kg for a period of 2 weeks), has been found to restore the 5-hydroxytryptamine levels in the mice's brains (Silvestro et al. 2021).

## Stress, anxiety, and depression

Anxiety and depression are closely associated with oxidative stress and overexpression of inflammatory molecules in the CNS. As discussed in the previous sections, CRMs have the potential to attenuate oxidative stress and inflammation in the brain, suggesting that they can help manage anxiety and depression. For instance, research conducted in male rats with inflammation induced by lipopolysaccharide, it was found that the use of metformin (50, 100, or 150 mg/ kg of body weight) was able to suppress depressive behavior in rats when analyzed through the behavioral tests (Kakhki et al. 2024). Another research confirmed the positive effects of resveratrol (40 and 80 mg/kg) in managing anxiety in rats when exposed to social isolation stress. This beneficial effect of resveratrol can be attributed to the reduction in inflammatory and oxidative stress biomarkers in the hippocampus (Baghaei Naeini et al. 2023). Yet another research postulated the effect of another CRM, rapamycin, in regulating anti-depressive activity in male Wistar rats induced with pentylenetetrazole. The rats showed improved behavior upon administration with rapamycin (Aghaie et al. 2021).

## Conclusion and future perspective

The influence of gut microbiota on the proper functioning of the brain has now become a well-established concept with a healthy diet becoming the need of the hour. The composition of the gut microbiota is very essential criterion when considering the type of influence it exerts on the brain since fluctuations in the composition of the microbiome lead to changes in levels of neurotransmitters and other metabolites secreted by the gut microbiota. This makes aging a key determinant of the bidirectional communication that exists between the gut and the brain, better known as GBA. With age, the microbial population changes in terms of both population size and species which can harm the GBA and disrupt normal functioning of the brain eventually leading to neurodegenerative diseases like AD and PD.

The neurons lose their tendency to clear the accumulated cellular waste, which results in toxic aggregate deposition and neuronal degeneration. This problem can however be dealt with by inducing autophagy in a dietary controlled manner. Dietary restriction concepts, for instance, intermittent fasting (IF) and calorie restriction (CR) gained popular interest because of their ability to control the calorie intake, thus triggering the autophagic pathway to remove accumulated cellular waste. However, these concepts have their limitations such as the need to follow a strict regime, lack of a generalized dose and effect study for such a vast population.

In this review, we have highlighted calorie restriction mimetics (CRMs) as a better alternative to produce the same dietary effect as generated by intermittent fasting or calorie restriction without having to make any major changes in our dietary intake. CRMs work through multiple pathways and can modulate the gut microbiota, for example, metformin, a well-established CRM can help metabolize glucose by activating the AMPK pathway. This indirectly helps maintain neuronal health by mTOR inhibition and clearance of toxic accumulates that can result from aging. Additionally, CRMs can positively impact gut microbiota by functional improvement of the intestinal barrier, gut hormone regulation, anti-inflammatory effects, etc. This paper summarizes various CRMs with their mechanism of action through which they help establish their respective effects, ultimately leading to enhanced autophagy and reduction of oxidative stress, the two main phenomena responsible for neurodegenerative disorders.

Although CRMs present a promising solution for regulating gut microbiota and its effects on the brain, there still exist gaps that need to be considered. As mentioned, the functioning of different CRMs occurs through a signaling cascade often involving multiple pathways and thus, there is a need to establish a clear cause-and-effect relationship triggered by a specific CRM. It is also to be noted that results obtained from preclinical studies still face the challenges of reproducing them during clinical trials. This could be speculated due to a lack of efficient strategies to deliver these CRMs to the target site. Additionally, the gut microbiota is highly transient and varies with age, gender, and geographic location, therefore, a detailed comprehensive study needs to be done to check for the potency of these CRMs in each of the research groups.

The incorporation of CRMs in the daily routine must be accompanied by a balanced diet, lacking which can lead to nutrient deficiencies. The use of CRMs might result in changes in metabolism and hormonal imbalance. This can prove a serious concern for individuals already suffering from diabetes, and hence they should receive proper consultation regarding ingestion of CRMs so that it does not interfere with blood sugar levels. Moreover, the fact that CRMs target such complex pathways could also lead to the possibility of them affecting these major pathways drastically when used for extended durations. Hence, the dosage and duration along with a balanced diet plan must be prepared before the administration of CRMs.To this end, research on CRMs has provided positive feedback to emerge as a neurotherapeutics candidate. With further research and clinical study, CRM's effect on enhancing health and longevity could be properly addressed and established.

## Data Availability

There is no data used in the research as described in this paper.

## References

[CR1] Aghaie F, Moradifar F, Hosseini A (2021) Rapamycin attenuates depression and anxiety-like behaviors through modulation of the NLRP3 pathway in pentylenetetrazole-kindled male Wistar rats. Fundam Clin Pharmacol 35:1045–1054. 10.1111/fcp.12689 33930202 10.1111/fcp.12689

[CR2] Ahmad S, Khan A, Ali W et al (2021) Fisetin rescues the mice brains against D-galactose-induced oxidative stress, neuroinflammation and memory impairment. Front Pharmacol. 10.3389/fphar.2021.612078 33716741 10.3389/fphar.2021.612078 PMC7947859

[CR3] Ahmad F, Javed M, Athar M, Shahzadi S (2023) Determination of affected brain regions at various stages of Alzheimer’s disease. Neurosci Res 192:77–82. 10.1016/j.neures.2023.01.010 36682693 10.1016/j.neures.2023.01.010

[CR4] Ahmadi S, Razazan A, Nagpal R et al (2020) Metformin reduces aging-related leaky gut and improves cognitive function by beneficially modulating gut microbiome/goblet cell/mucin axis. J Gerontol A Biol Sci Med Sci 75:e9–e21. 10.1093/gerona/glaa056 32129462 10.1093/gerona/glaa056 PMC7302182

[CR5] Al-Qudah M, Anderson CD, Mahavadi S et al (2014) Brain-derived neurotrophic factor enhances cholinergic contraction of longitudinal muscle of rabbit intestine via activation of phospholipase C. Am J Physiol Gastrointest Liver Physiol 306:G328-337. 10.1152/ajpgi.00203.2013 24356881 10.1152/ajpgi.00203.2013 PMC3920121

[CR6] Alshehri AS, El-Kott AF, El-Kenawy AE et al (2022) The ameliorative effect of kaempferol against CdCl2- mediated renal damage entails activation of Nrf2 and inhibition of NF-kB. Environ Sci Pollut Res Int 29:57591–57602. 10.1007/s11356-022-19876-7 35355181 10.1007/s11356-022-19876-7

[CR7] Amigo I, Menezes-Filho SL, Luévano-Martínez LA et al (2017) Caloric restriction increases brain mitochondrial calcium retention capacity and protects against excitotoxicity. Aging Cell 16:73–81. 10.1111/acel.12527 27619151 10.1111/acel.12527 PMC5242290

[CR8] Antony PMA, Diederich NJ, Krüger R, Balling R (2013) The hallmarks of Parkinson’s disease. FEBS J 280:5981–5993. 10.1111/febs.12335 23663200 10.1111/febs.12335

[CR9] Apple DM, Solano-Fonseca R, Kokovay E (2017) Neurogenesis in the aging brain. Biochem Pharmacol 141:77–85. 10.1016/j.bcp.2017.06.116 28625813 10.1016/j.bcp.2017.06.116

[CR10] Arya JK, Kumar R, Singh A et al (2023) Acarbose, an α-glucosidase inhibitor, maintains altered redox homeostasis during aging by targeting glucose metabolism in rat erythrocytes. Rejuvenation Res 26:21–31. 10.1089/rej.2022.0032 36524249 10.1089/rej.2022.0032

[CR11] Atayik MC, Çakatay U (2023) Redox signaling and modulation in ageing. Biogerontology 24:603–608. 10.1007/s10522-023-10055-w 37535201 10.1007/s10522-023-10055-w

[CR12] Athari Nik Azm S, Djazayeri A, Safa M et al (2018) Lactobacilli and bifidobacteria ameliorate memory and learning deficits and oxidative stress in β-amyloid (1–42) injected rats. Appl Physiol Nutr Metab 43:718–726. 10.1139/apnm-2017-0648 29462572 10.1139/apnm-2017-0648

[CR13] Baghaei Naeini F, Hassanpour S, Asghari A (2023) Resveratrol exerts anxiolytic-like effects through anti-inflammatory and antioxidant activities in rats exposed to chronic social isolation. Behav Brain Res 438:114201. 10.1016/j.bbr.2022.114201 36334782 10.1016/j.bbr.2022.114201

[CR14] Bailey CJ (2017) Metformin: historical overview. Diabetologia 60:1566–1576. 10.1007/s00125-017-4318-z 28776081 10.1007/s00125-017-4318-z

[CR15] Barbara G, Stanghellini V, Brandi G et al (2005) Interactions between commensal bacteria and gut sensorimotor function in health and disease. Am J Gastroenterol 100:2560–2568. 10.1111/j.1572-0241.2005.00230.x 16279914 10.1111/j.1572-0241.2005.00230.x

[CR16] Barini E, Antico O, Zhao Y et al (2016) Metformin promotes tau aggregation and exacerbates abnormal behavior in a mouse model of tauopathy. Mol Neurodegener 11:16. 10.1186/s13024-016-0082-7 26858121 10.1186/s13024-016-0082-7 PMC4746897

[CR17] Baroni MD, Colombo S, Libens O et al (2020) In S. cerevisiae hydroxycitric acid antagonizes chronological aging and apoptosis regardless of citrate lyase. Apoptosis 25:686–696. 10.1007/s10495-020-01625-1 32666259 10.1007/s10495-020-01625-1 PMC7527365

[CR18] Baur JA, Pearson KJ, Price NL et al (2006) Resveratrol improves health and survival of mice on a high-calorie diet. Nature 444:337–342. 10.1038/nature05354 17086191 10.1038/nature05354 PMC4990206

[CR19] Beltz BS, Tlusty MF, Benton JL, Sandeman DC (2007) Omega-3 fatty acids upregulate adult neurogenesis. Neurosci Lett 415:154–158. 10.1016/j.neulet.2007.01.010 17240063 10.1016/j.neulet.2007.01.010 PMC1892224

[CR20] Bonaz B, Sinniger V, Pellissier S (2017) Vagus nerve stimulation: a new promising therapeutic tool in inflammatory bowel disease. J Intern Med 282:46–63. 10.1111/joim.12611 28421634 10.1111/joim.12611

[CR21] Borrego-Ruiz A, Borrego JJ (2024) Human gut microbiome, diet, and mental disorders. Int Microbiol. 10.1007/s10123-024-00518-6 38561477 10.1007/s10123-024-00518-6 PMC11775079

[CR22] Borsini A, Stangl D, Jeffries AR et al (2020) The role of omega-3 fatty acids in preventing glucocorticoid-induced reduction in human hippocampal neurogenesis and increase in apoptosis. Transl Psychiatry 10:219. 10.1038/s41398-020-00908-0 32636362 10.1038/s41398-020-00908-0 PMC7341841

[CR23] Cabreiro F, Au C, Leung K-Y et al (2013) Metformin retards aging in C. elegans by altering microbial folate and methionine metabolism. Cell 153:228–239. 10.1016/j.cell.2013.02.035 23540700 10.1016/j.cell.2013.02.035 PMC3898468

[CR24] Cai J, Rimal B, Jiang C et al (2022) Bile acid metabolism and signaling, the microbiota, and metabolic disease. Pharmacol Ther 237:108238. 10.1016/j.pharmthera.2022.108238 35792223 10.1016/j.pharmthera.2022.108238

[CR25] Cantó C, Jiang LQ, Deshmukh AS et al (2010) Interdependence of AMPK and SIRT1 for metabolic adaptation to fasting and exercise in skeletal muscle. Cell Metab 11:213–219. 10.1016/j.cmet.2010.02.006 20197054 10.1016/j.cmet.2010.02.006 PMC3616265

[CR26] Chelakkot C, Ghim J, Ryu SH (2018) Mechanisms regulating intestinal barrier integrity and its pathological implications. Exp Mol Med 50:1–9. 10.1038/s12276-018-0126-x 30115904 10.1038/s12276-018-0126-x PMC6095905

[CR27] Chen M, Yi L, Zhang Y et al (2016) Resveratrol attenuates trimethylamine-N-oxide (TMAO)-induced atherosclerosis by regulating TMAO synthesis and bile acid metabolism via remodeling of the gut microbiota. Mbio 7:e02210-02215. 10.1128/mBio.02210-15 27048804 10.1128/mBio.02210-15 PMC4817264

[CR28] Chen Y, Xu J, Chen Y (2021a) Regulation of neurotransmitters by the gut microbiota and effects on cognition in neurological disorders. Nutrients 13:2099. 10.3390/nu13062099 34205336 10.3390/nu13062099 PMC8234057

[CR29] Chen Y, Zhou J, Wang L (2021b) Role and mechanism of gut microbiota in human disease. Front Cell Infect Microbiol. 10.3389/fcimb.2021.625913 33816335 10.3389/fcimb.2021.625913 PMC8010197

[CR30] Chiang M-C, Cheng Y-C, Chen S-J et al (2016) Metformin activation of AMPK-dependent pathways is neuroprotective in human neural stem cells against Amyloid-beta-induced mitochondrial dysfunction. Exp Cell Res 347:322–331. 10.1016/j.yexcr.2016.08.013 27554603 10.1016/j.yexcr.2016.08.013

[CR31] Chuengsamarn S, Rattanamongkolgul S, Phonrat B et al (2014) Reduction of atherogenic risk in patients with type 2 diabetes by curcuminoid extract: a randomized controlled trial. J Nutr Biochem 25:144–150. 10.1016/j.jnutbio.2013.09.013 24445038 10.1016/j.jnutbio.2013.09.013

[CR32] Clarke G, Grenham S, Scully P et al (2013) The microbiome-gut-brain axis during early life regulates the hippocampal serotonergic system in a sex-dependent manner. Mol Psychiatry 18:666–673. 10.1038/mp.2012.77 22688187 10.1038/mp.2012.77

[CR33] Deverman BE, Patterson PH (2009) Cytokines and CNS development. Neuron 64:61–78. 10.1016/j.neuron.2009.09.002 19840550 10.1016/j.neuron.2009.09.002

[CR34] Doan KV, Ko CM, Kinyua AW et al (2015) Gallic acid regulates body weight and glucose homeostasis through AMPK activation. Endocrinology 156:157–168. 10.1210/en.2014-1354 25356824 10.1210/en.2014-1354

[CR35] Espinoza JL, Trung LQ, Inaoka PT et al (2017) The repeated administration of resveratrol has measurable effects on circulating T-cell subsets in humans. Oxid Med Cell Longev 2017:6781872. 10.1155/2017/6781872 28546852 10.1155/2017/6781872 PMC5435979

[CR36] Ferguson JJA, Abbott KA, Garg ML (2021) Anti-inflammatory effects of oral supplementation with curcumin: a systematic review and meta-analysis of randomized controlled trials. Nutr Rev 79:1043–1066. 10.1093/nutrit/nuaa114 34378053 10.1093/nutrit/nuaa114

[CR37] Ferreiro AL, Choi J, Ryou J et al (2023) Gut microbiome composition may be an indicator of preclinical Alzheimer’s disease. Sci Transl Med 15:eabo2984. 10.1126/scitranslmed.abo2984 37315112 10.1126/scitranslmed.abo2984 PMC10680783

[CR38] Foudah AI, Devi S, Alam A et al (2023) Anticholinergic effect of resveratrol with vitamin E on scopolamine-induced Alzheimer’s disease in rats: Mechanistic approach to prevent inflammation. Front Pharmacol 14:1115721. 10.3389/fphar.2023.1115721 36817151 10.3389/fphar.2023.1115721 PMC9932024

[CR39] Fusco W, Lorenzo MB, Cintoni M et al (2023) Short-chain fatty-acid-producing bacteria: key components of the human gut microbiota. Nutrients 15:2211. 10.3390/nu15092211 37432351 10.3390/nu15092211 PMC10180739

[CR40] Gabandé-Rodríguez E, Gómez de las Heras MM, Mittelbrunn M (2020) Control of inflammation by calorie restriction mimetics: on the crossroad of autophagy and mitochondria. Cells 9:82. 10.3390/cells9010082 10.3390/cells9010082 PMC7017321 31905682

[CR41] Gabarró-Solanas R, Davaatseren A, Kleifeld J et al (2023) Adult neural stem cells and neurogenesis are resilient to intermittent fasting. EMBO Rep 24:e57268. 10.15252/embr.202357268 37987220 10.15252/embr.202357268 PMC10702802

[CR42] Ghafouri-Fard S, Shoorei H, Khanbabapour Sasi A et al (2021) The impact of the phytotherapeutic agent quercetin on expression of genes and activity of signaling pathways. Biomed Pharmacother 141:111847. 10.1016/j.biopha.2021.111847 34198048 10.1016/j.biopha.2021.111847

[CR43] Glick D, Barth S, Macleod KF (2010) Autophagy: cellular and molecular mechanisms. J Pathol 221:3–12. 10.1002/path.2697 20225336 10.1002/path.2697 PMC2990190

[CR44] Greer EL, Banko MR, Brunet A (2009) AMP-activated protein kinase and FoxO transcription factors in dietary restriction-induced longevity. Ann NY Acad Sci 1170:688–692. 10.1111/j.1749-6632.2009.04019.x 19686213 10.1111/j.1749-6632.2009.04019.x PMC2814416

[CR45] Gregor A, Pignitter M, Trajanoski S et al (2021) Microbial contribution to the caloric restriction-triggered regulation of the intestinal levels of glutathione transferases, taurine, and bile acid. Gut Microbes 13:1992236. 10.1080/19490976.2021.1992236 34693866 10.1080/19490976.2021.1992236 PMC8547879

[CR46] Gualdoni GA, Kovarik JJ, Hofer J et al (2014) Resveratrol enhances TNF-α production in human monocytes upon bacterial stimulation. Biochimica Et Biophysica Acta (BBA)—Gen Subj 1840:95–105 10.1016/j.bbagen.2013.09.009 24035785

[CR47] Gudden J, Arias Vasquez A, Bloemendaal M (2021) The effects of intermittent fasting on brain and cognitive function. Nutrients 13:3166. 10.3390/nu13093166 34579042 10.3390/nu13093166 PMC8470960

[CR48] Gué M, Peeters T, Depoortere I et al (1989) Stress-induced changes in gastric emptying, postprandial motility, and plasma gut hormone levels in dogs. Gastroenterology 97:1101–1107. 10.1016/0016-5085(89)91678-8 2571543 10.1016/0016-5085(89)91678-8

[CR49] Gue M, Junien JL, Bueno L (1991) Conditioned emotional response in rats enhances colonic motility through the central release of corticotropin-releasing factor. Gastroenterology 100:964–970. 10.1016/0016-5085(91)90270-u 2001832 10.1016/0016-5085(91)90270-u

[CR50] Guo Z, Lee J, Lane M, Mattson MP (2001) Iodoacetate protects hippocampal neurons against excitotoxic and oxidative injury: involvement of heat-shock proteins and Bcl-2. J Neurochem 79:361–370. 10.1046/j.1471-4159.2001.00564.x 11677264 10.1046/j.1471-4159.2001.00564.x

[CR51] Halagappa VKM, Guo Z, Pearson M et al (2007) Intermittent fasting and caloric restriction ameliorate age-related behavioral deficits in the triple-transgenic mouse model of Alzheimer’s disease. Neurobiol Dis 26:212–220. 10.1016/j.nbd.2006.12.019 17306982 10.1016/j.nbd.2006.12.019

[CR52] Hariri M, Haghighatdoost F (2018) Effect of curcumin on anthropometric measures: a systematic review on randomized clinical trials. J Am Coll Nutr 37:215–222. 10.1080/07315724.2017.1392263 29313748 10.1080/07315724.2017.1392263

[CR53] Hasan N, Yang H (2019) Factors affecting the composition of the gut microbiota, and its modulation. PeerJ 7:e7502. 10.7717/peerj.7502 31440436 10.7717/peerj.7502 PMC6699480

[CR54] Hassani B, Goshtasbi G, Nooraddini S, Firouzabadi N (2022) Pharmacological approaches to decelerate aging: a promising path. Oxid Med Cell Longev. 10.1155/2022/4201533 35860429 10.1155/2022/4201533 PMC9293537

[CR55] Heijtz RD, Wang S, Anuar F et al (2011) Normal gut microbiota modulates brain development and behavior. Proc Natl Acad Sci 108:3047–3052. 10.1073/pnas.1010529108 21282636 10.1073/pnas.1010529108 PMC3041077

[CR56] Hoffmann GE, Andres H, Weiss L et al (1980) Properties and organ distribution of ATP citrate (pro-3S)-lyase. Biochim Biophys Acta 620:151–158. 10.1016/0005-2760(80)90194-0 7417478 10.1016/0005-2760(80)90194-0

[CR57] Homolak J (2023a) Chapter eleven—targeting the microbiota-mitochondria crosstalk in neurodegeneration with senotherapeutics. In: Çakatay U, Atayik MC (eds) Advances in protein chemistry and structural biology. Academic Press, Cambridge, pp 339–383 10.1016/bs.apcsb.2023.02.018 37437983

[CR58] Homolak J (2023b) Gastrointestinal redox homeostasis in ageing. Biogerontology 24:741–752. 10.1007/s10522-023-10049-8 37436501 10.1007/s10522-023-10049-8

[CR59] Homolak J, De Busscher J, Zambrano-Lucio M et al (2023) Altered secretion, constitution, and functional properties of the gastrointestinal mucus in a rat model of sporadic Alzheimer’s disease. ACS Chem Neurosci 14:2667–2682. 10.1021/acschemneuro.3c00223 37477640 10.1021/acschemneuro.3c00223 PMC10401635

[CR60] Hou K, Wu Z-X, Chen X-Y et al (2022) Microbiota in health and diseases. Signal Transduct Target Ther 7:135. 10.1038/s41392-022-00974-4 35461318 10.1038/s41392-022-00974-4 PMC9034083

[CR61] Howitz KT, Bitterman KJ, Cohen HY et al (2003) Small molecule activators of sirtuins extend *Saccharomyces cerevisiae* lifespan. Nature 425:191–196. 10.1038/nature01960 12939617 10.1038/nature01960

[CR62] Hsuchou H, Pan W, Kastin AJ (2013) Fibroblast growth factor 19 entry into brain. Fluids Barriers CNS 10:32. 10.1186/2045-8118-10-32 24176017 10.1186/2045-8118-10-32 PMC3818657

[CR63] Huang J, Chen L, Xue B et al (2016) Different flavonoids can shape unique gut microbiota profile in vitro. J Food Sci 81:H2273-2279. 10.1111/1750-3841.13411 27472307 10.1111/1750-3841.13411

[CR64] Husebye E, Hellström PM, Sundler F et al (2001) Influence of microbial species on small intestinal myoelectric activity and transit in germ-free rats. Am J Physiol Gastrointest Liver Physiol 280:G368-380. 10.1152/ajpgi.2001.280.3.G368 11171619 10.1152/ajpgi.2001.280.3.G368

[CR65] Ingram DK, Roth GS (2011) Glycolytic inhibition as a strategy for developing calorie restriction mimetics. Exp Gerontol 46:148–154. 10.1016/j.exger.2010.12.001 21167272 10.1016/j.exger.2010.12.001

[CR66] Ingram DK, Roth GS (2015) Calorie restriction mimetics: can you have your cake and eat it, too? Ageing Res Rev 20:46–62. 10.1016/j.arr.2014.11.005 25530568 10.1016/j.arr.2014.11.005

[CR67] Isaev NK, Stelmashook EV, Genrikhs EE (2019) Neurogenesis and brain aging. Rev Neurosci 30:573–580. 10.1515/revneuro-2018-0084 30763272 10.1515/revneuro-2018-0084

[CR68] Jandhyala SM, Talukdar R, Subramanyam C et al (2015) Role of the normal gut microbiota. World J Gastroenterol 21:8787–8803. 10.3748/wjg.v21.i29.8787 26269668 10.3748/wjg.v21.i29.8787 PMC4528021

[CR69] Ji L-L, Sheng Y-C, Zheng Z-Y et al (2015) The involvement of p62-Keap1-Nrf2 antioxidative signaling pathway and JNK in the protection of natural flavonoid quercetin against hepatotoxicity. Free Radic Biol Med 85:12–23. 10.1016/j.freeradbiomed.2015.03.035 25881548 10.1016/j.freeradbiomed.2015.03.035

[CR70] Ji S, Wang L, Li L (2019) Effect of metformin on short-term high-fat diet-induced weight gain and anxiety-like behavior and the gut microbiota. Front Endocrinol 10:704. 10.3389/fendo.2019.00704 10.3389/fendo.2019.00704 PMC6813541 31681174

[CR71] Jones RM, Neish AS (2017) Redox signaling mediated by the gut microbiota. Free Radical Biol Med 105:41–47. 10.1016/j.freeradbiomed.2016.10.495 27989756 10.1016/j.freeradbiomed.2016.10.495

[CR72] Jones MP, Dilley JB, Drossman D, Crowell MD (2006) Brain-gut connections in functional GI disorders: anatomic and physiologic relationships. Neurogastroenterol Motil 18:91–103. 10.1111/j.1365-2982.2005.00730.x 16420287 10.1111/j.1365-2982.2005.00730.x

[CR73] Ju Y, Tam KY (2022) Pathological mechanisms and therapeutic strategies for Alzheimer’s disease. Neural Regen Res 17:543–549. 10.4103/1673-5374.320970 34380884 10.4103/1673-5374.320970 PMC8504384

[CR74] Kakhki FSH, Asghari A, Bardaghi Z et al (2024) The antidiabetic drug metformin attenuated depressive and anxietylike behaviors and oxidative stress in the brain in a rodent model of inflammation induced by lipopolysaccharide in male rats. Endocr Metabol Immun Disord—Drug Targets 24:1525–1537. 10.2174/0118715303275039231228065050 10.2174/0118715303275039231228065050 38284725

[CR75] Kang Y, Sun Y, Li T, Ren Z (2020) Garcinol protects against cerebral ischemia-reperfusion injury in vivo and in vitro by inhibiting inflammation and oxidative stress. Mol Cell Probes 54:101672. 10.1016/j.mcp.2020.101672 33186709 10.1016/j.mcp.2020.101672

[CR76] Kapoor MP, Sugita M, Fukuzawa Y, Okubo T (2017) Physiological effects of epigallocatechin-3-gallate (EGCG) on energy expenditure for prospective fat oxidation in humans: a systematic review and meta-analysis. J Nutr Biochem 43:1–10. 10.1016/j.jnutbio.2016.10.013 27883924 10.1016/j.jnutbio.2016.10.013

[CR77] Kelly JR, Kennedy PJ, Cryan JF et al (2015) Breaking down the barriers: the gut microbiome, intestinal permeability and stress-related psychiatric disorders. Front Cell Neurosci 9:392. 10.3389/fncel.2015.00392 26528128 10.3389/fncel.2015.00392 PMC4604320

[CR78] Khan N, Afaq F, Khusro FH et al (2012) Dual inhibition of pi3k/akt and mtor signaling in human non-small cell lung cancer cells by a dietary flavonoid fisetin. Int J Cancer 130:1695–1705. 10.1002/ijc.26178 21618507 10.1002/ijc.26178 PMC3267899

[CR79] Khan A, Park JS, Kang MH et al (2023) Caffeic acid, a polyphenolic micronutrient rescues mice brains against aβ-induced neurodegeneration and memory impairment. Antioxidants 12:1284. 10.3390/antiox12061284 37372012 10.3390/antiox12061284 PMC10295243

[CR80] Kim N, Jeon SH, Ju IG et al (2021) Transplantation of gut microbiota derived from Alzheimer’s disease mouse model impairs memory function and neurogenesis in C57BL/6 mice. Brain Behav Immun 98:357–365. 10.1016/j.bbi.2021.09.002 34500036 10.1016/j.bbi.2021.09.002

[CR81] Kirpichnikov D, McFarlane SI, Sowers JR (2002) Metformin: an update. Ann Intern Med 137:25–33. 10.7326/0003-4819-137-1-200207020-00009 12093242 10.7326/0003-4819-137-1-200207020-00009

[CR82] Klempin F, Kempermann G (2007) Adult hippocampal neurogenesis and aging. Eur Arch Psychiatry Clin Neurosci 257:271–280. 10.1007/s00406-007-0731-5 17401726 10.1007/s00406-007-0731-5

[CR83] Kma L, Baruah TJ (2022) The interplay of ROS and the PI3K/Akt pathway in autophagy regulation. Biotechnol Appl Biochem 69:248–264. 10.1002/bab.2104 33442914 10.1002/bab.2104

[CR84] Kubota N, Yano W, Kubota T et al (2007) Adiponectin stimulates AMP-activated protein kinase in the hypothalamus and increases food intake. Cell Metab 6:55–68. 10.1016/j.cmet.2007.06.003 17618856 10.1016/j.cmet.2007.06.003

[CR85] Kumar R, Saraswat K, Rizvi SI (2020) 2 –Deoxy—d-glucose at chronic low dose acts as a caloric restriction mimetic through a mitohormetic induction of ROS in the brain of accelerated senescence model of rat. Arch Gerontol Geriatr 90:104133. 10.1016/j.archger.2020.104133 32559563 10.1016/j.archger.2020.104133

[CR86] Lane MA, Ingram DK, Roth GS (1998) 2-Deoxy-d-glucose feeding in rats mimics physiologic effects of calorie restriction. J Anti Aging Med 1:327–337. 10.1089/rej.1.1998.1.327

[CR87] Largo R, Alvarez-Soria MA, Díez-Ortego I et al (2003) Glucosamine inhibits IL-1beta-induced NFkappaB activation in human osteoarthritic chondrocytes. Osteoarthr Cartil 11:290–298. 10.1016/s1063-4584(03)00028-1 10.1016/s1063-4584(03)00028-1 12681956

[CR88] Lee J, Duan W, Long JM et al (2000) Dietary restriction increases the number of newly generated neural cells, and induces BDNF expression, in the dentate gyrus of rats. J Mol Neurosci 15:99–108. 10.1385/JMN:15:2:99 11220789 10.1385/JMN:15:2:99

[CR89] Lee YS, Kim W, Kim K et al (2006) Berberine, a natural plant product, activates AMP-activated protein kinase with beneficial metabolic effects in diabetic and insulin-resistant states. Diabetes 55:2256–2264. 10.2337/db06-0006 16873688 10.2337/db06-0006

[CR90] Liénard C, Pintart A, Bomont P (2024) Neuronal autophagy: regulations and implications in health and disease. Cells 13:103. 10.3390/cells13010103 38201307 10.3390/cells13010103 PMC10778363

[CR91] Likhitwitayawuid K (2021) Oxyresveratrol: sources, productions, biological activities, pharmacokinetics, and delivery systems. Molecules 26:4212. 10.3390/molecules26144212 34299485 10.3390/molecules26144212 PMC8307110

[CR92] Liu Y, Tang G, Li Y et al (2014) Metformin attenuates blood-brain barrier disruption in mice following middle cerebral artery occlusion. J Neuroinflammation 11:177. 10.1186/s12974-014-0177-4 25315906 10.1186/s12974-014-0177-4 PMC4201919

[CR93] Liu S, Marcelin G, Blouet C et al (2018) A gut-brain axis regulating glucose metabolism mediated by bile acids and competitive fibroblast growth factor actions at the hypothalamus. Mol Metab 8:37–50. 10.1016/j.molmet.2017.12.003 29290621 10.1016/j.molmet.2017.12.003 PMC5985052

[CR94] Liu C, Yang S-Y, Wang L, Zhou F (2022) The gut microbiome: implications for neurogenesis and neurological diseases. Neural Regen Res 17:53–58. 10.4103/1673-5374.315227 34100427 10.4103/1673-5374.315227 PMC8451566

[CR95] Lozupone CA, Stombaugh JI, Gordon JI et al (2012) Diversity, stability and resilience of the human gut microbiota. Nature 489:220–230. 10.1038/nature11550 22972295 10.1038/nature11550 PMC3577372

[CR96] Lu M, Chen H, Nie F et al (2020) The potential role of metformin in the treatment of Parkinson’s disease. J Bio-X Res 3:27. 10.1097/JBR.0000000000000055

[CR97] Ma Y, Shi Y, Wu Q, Ma W (2021) Epigallocatechin-3-gallate alleviates vanadium-induced reduction of antioxidant capacity via keap1-Nrf2-smaf pathway in the liver, kidney, and ovary of laying hens. Biol Trace Elem Res 199:2707–2716. 10.1007/s12011-020-02398-z 33405082 10.1007/s12011-020-02398-z

[CR98] Macfarlane S, Dillon JF (2007) Microbial biofilms in the human gastrointestinal tract. J Appl Microbiol 102:1187–1196. 10.1111/j.1365-2672.2007.03287.x 17448154 10.1111/j.1365-2672.2007.03287.x

[CR99] Madeo F, Carmona-Gutierrez D, Hofer SJ, Kroemer G (2019a) Caloric restriction mimetics against age-associated disease: targets, mechanisms, and therapeutic potential. Cell Metab 29:592–610. 10.1016/j.cmet.2019.01.018 30840912 10.1016/j.cmet.2019.01.018

[CR100] Mandrioli J, D’Amico R, Zucchi E et al (2023) Randomized, double-blind, placebo-controlled trial of rapamycin in amyotrophic lateral sclerosis. Nat Commun 14:4970. 10.1038/s41467-023-40734-8 37591957 10.1038/s41467-023-40734-8 PMC10435464

[CR101] Mariño G, Pietrocola F, Eisenberg T et al (2014) Regulation of autophagy by cytosolic acetyl-coenzyme A. Mol Cell 53:710–725. 10.1016/j.molcel.2014.01.016 24560926 10.1016/j.molcel.2014.01.016

[CR102] Maswood N, Young J, Tilmont E et al (2004) Caloric restriction increases neurotrophic factor levels and attenuates neurochemical and behavioral deficits in a primate model of Parkinson’s disease. Proc Natl Acad Sci USA 101:18171–18176. 10.1073/pnas.0405831102 15604149 10.1073/pnas.0405831102 PMC539733

[CR103] Mayor E (2023) Neurotrophic effects of intermittent fasting, calorie restriction and exercise: a review and annotated bibliography. Front Aging 4:1161814. 10.3389/fragi.2023.1161814 37334045 10.3389/fragi.2023.1161814 PMC10273285

[CR104] McGuinness AJ, Davis JA, Dawson SL et al (2022) A systematic review of gut microbiota composition in observational studies of major depressive disorder, bipolar disorder and schizophrenia. Mol Psychiatry 27:1920–1935. 10.1038/s41380-022-01456-3 35194166 10.1038/s41380-022-01456-3 PMC9126816

[CR105] Menozzi E, Schapira AHV (2024) The gut microbiota in parkinson disease: interactions with drugs and potential for therapeutic applications. CNS Drugs 38:315–331. 10.1007/s40263-024-01073-4 38570412 10.1007/s40263-024-01073-4 PMC11026199

[CR106] Michan S, Sinclair D (2007) Sirtuins in mammals: insights into their biological function. Biochem J 404:1–13. 10.1042/BJ20070140 17447894 10.1042/BJ20070140 PMC2753453

[CR107] Mirzaei R, Bouzari B, Hosseini-Fard SR et al (2021) Role of microbiota-derived short-chain fatty acids in nervous system disorders. Biomed Pharmacother 139:111661. 10.1016/j.biopha.2021.111661 34243604 10.1016/j.biopha.2021.111661

[CR108] Möhle L, Mattei D, Heimesaat MM et al (2016) Ly6C(hi) monocytes provide a link between antibiotic-induced changes in gut microbiota and adult hippocampal neurogenesis. Cell Rep 15:1945–1956. 10.1016/j.celrep.2016.04.074 27210745 10.1016/j.celrep.2016.04.074

[CR109] Moran-Ramos S, Lopez-Contreras BE, Villarruel-Vazquez R et al (2020) Environmental and intrinsic factors shaping gut microbiota composition and diversity and its relation to metabolic health in children and early adolescents: a population-based study. Gut Microbes 11:900–917. 10.1080/19490976.2020.1712985 31973685 10.1080/19490976.2020.1712985 PMC7524342

[CR110] Morselli E, Maiuri MC, Markaki M et al (2010) Caloric restriction and resveratrol promote longevity through the sirtuin-1-dependent induction of autophagy. Cell Death Dis 1:e10. 10.1038/cddis.2009.8 21364612 10.1038/cddis.2009.8 PMC3032517

[CR111] Morselli E, Mariño G, Bennetzen MV et al (2011) Spermidine and resveratrol induce autophagy by distinct pathways converging on the acetylproteome. J Cell Biol 192:615–629. 10.1083/jcb.201008167 21339330 10.1083/jcb.201008167 PMC3044119

[CR112] Moussa C, Hebron M, Huang X et al (2017) Resveratrol regulates neuro-inflammation and induces adaptive immunity in Alzheimer’s disease. J Neuroinflammation 14:1. 10.1186/s12974-016-0779-0 28086917 10.1186/s12974-016-0779-0 PMC5234138

[CR113] Navarro SL, White E, Kantor ED et al (2015) Randomized trial of glucosamine and chondroitin supplementation on inflammation and oxidative stress biomarkers and plasma proteomics profiles in healthy humans. PLoS ONE 10:e0117534. 10.1371/journal.pone.0117534 25719429 10.1371/journal.pone.0117534 PMC4342228

[CR114] Papila B, Karimova A, Onaran I (2024) Altered lactate/pyruvate ratio may be responsible for aging-associated intestinal barrier dysfunction in male rats. Biogerontology 25:679–689. 10.1007/s10522-024-10102-0 38619668 10.1007/s10522-024-10102-0 PMC11217102

[CR115] Park D-J, Kang J-B, Koh P-O (2024) Epigallocatechin gallate improves neuronal damage in animal model of ischemic stroke and glutamate-exposed neurons via modulation of hippocalcin expression. PLoS ONE 19:e0299042. 10.1371/journal.pone.0299042 38427657 10.1371/journal.pone.0299042 PMC10906901

[CR116] Pekar T, Bruckner K, Pauschenwein-Frantsich S et al (2021) The positive effect of spermidine in older adults suffering from dementia: first results of a 3-month trial. Wien Klin Wochenschr 133:484–491. 10.1007/s00508-020-01758-y 33211152 10.1007/s00508-020-01758-y PMC8116233

[CR117] Peng Y, Jin H, Xue Y-H et al (2023) Current and future therapeutic strategies for Alzheimer’s disease: an overview of drug development bottlenecks. Front Aging Neurosci 15:1206572. 10.3389/fnagi.2023.1206572 37600514 10.3389/fnagi.2023.1206572 PMC10438465

[CR118] Perlman JM (2002) Cognitive and behavioral deficits in premature graduates of intensive care. Clin Perinatol 29:779–797. 10.1016/s0095-5108(02)00051-9 12516746 10.1016/s0095-5108(02)00051-9

[CR119] Phung OJ, Baker WL, Matthews LJ et al (2010) Effect of green tea catechins with or without caffeine on anthropometric measures: a systematic review and meta-analysis. Am J Clin Nutr 91:73–81. 10.3945/ajcn.2009.28157 19906797 10.3945/ajcn.2009.28157

[CR120] Picca A, Fracasso F, Pesce V et al (2013) Age- and calorie restriction-related changes in rat brain mitochondrial DNA and TFAM binding. Age (dordr) 35:1607–1620. 10.1007/s11357-012-9465-z 22945739 10.1007/s11357-012-9465-z PMC3776104

[CR121] Pietrocola F, Castoldi F, Maiuri MC, Kroemer G (2018) Aspirin—another caloric-restriction mimetic. Autophagy 14:1162–1163. 10.1080/15548627.2018.1454810 29929449 10.1080/15548627.2018.1454810 PMC6103658

[CR122] Portero-Tresserra M, Rojic-Becker D, Vega-Carbajal C et al (2020) Caloric restriction modulates the monoaminergic system and metabolic hormones in aged rats. Sci Rep 10:19299. 10.1038/s41598-020-76219-7 33168891 10.1038/s41598-020-76219-7 PMC7653031

[CR123] Price NL, Gomes AP, Ling AJY et al (2012) SIRT1 is required for AMPK activation and the beneficial effects of resveratrol on mitochondrial function. Cell Metab 15:675–690. 10.1016/j.cmet.2012.04.003 22560220 10.1016/j.cmet.2012.04.003 PMC3545644

[CR124] Prvulovic M, Todorovic S, Milanovic D et al (2022) Calorie restriction changes the anxiety-like behaviour of ageing male Wistar rats in an onset- and duration-dependent manner. Mech Ageing Dev 204:111666. 10.1016/j.mad.2022.111666 35331743 10.1016/j.mad.2022.111666

[CR125] Qin W, Zhao W, Ho L et al (2008) Regulation of forkhead transcription factor FoxO3a contributes to calorie restriction-induced prevention of Alzheimer’s disease-type amyloid neuropathology and spatial memory deterioration. Ann NY Acad Sci 1147:335–347. 10.1196/annals.1427.024 19076455 10.1196/annals.1427.024 PMC2605640

[CR126] Rattan SIS (2024) Seven knowledge gaps in modern biogerontology. Biogerontology 25:1–8. 10.1007/s10522-023-10089-0 38206540 10.1007/s10522-023-10089-0

[CR127] Rege SD, Geetha T, Griffin GD et al (2014a) Neuroprotective effects of resveratrol in Alzheimer disease pathology. Front Aging Neurosci. 10.3389/fnagi.2014.00218 25309423 10.3389/fnagi.2014.00218 PMC4161050

[CR128] Rha C-S, Seong H, Jung YS et al (2019) Stability and fermentability of green tea flavonols in in-vitro-simulated gastrointestinal digestion and human fecal fermentation. Int J Mol Sci 20:5890. 10.3390/ijms20235890 31771257 10.3390/ijms20235890 PMC6928927

[CR129] Rhee SH, Pothoulakis C, Mayer EA (2009) Principles and clinical implications of the brain-gut-enteric microbiota axis. Nat Rev Gastroenterol Hepatol 6:306–314. 10.1038/nrgastro.2009.35 19404271 10.1038/nrgastro.2009.35 PMC3817714

[CR130] Ribeiro MF, Santos AA, Afonso MB et al (2020) Diet-dependent gut microbiota impacts on adult neurogenesis through mitochondrial stress modulation. Brain Commun 2:fcaa165. 10.1093/braincomms/fcaa165 33426525 10.1093/braincomms/fcaa165 PMC7780462

[CR131] Rinninella E, Raoul P, Cintoni M et al (2019) What is the healthy gut microbiota composition? A changing ecosystem across age, environment, diet, and diseases. Microorganisms 7:14. 10.3390/microorganisms7010014 30634578 10.3390/microorganisms7010014 PMC6351938

[CR132] Romano S, Savva GM, Bedarf JR et al (2021) Meta-analysis of the Parkinson’s disease gut microbiome suggests alterations linked to intestinal inflammation. Npj Parkinsons Dis 7:1–13. 10.1038/s41531-021-00156-z 33692356 10.1038/s41531-021-00156-z PMC7946946

[CR133] Roth W, Zadeh K, Vekariya R et al (2021) Tryptophan metabolism and gut-brain homeostasis. Int J Mol Sci 22:2973. 10.3390/ijms22062973 33804088 10.3390/ijms22062973 PMC8000752

[CR134] Saha S, Buttari B, Panieri E et al (2020) An overview of Nrf2 signaling pathway and its role in inflammation. Molecules 25:5474. 10.3390/molecules25225474 33238435 10.3390/molecules25225474 PMC7700122

[CR135] Sawda C, Moussa C, Turner RS (2017) Resveratrol for Alzheimer’s disease. Ann NY Acad Sci 1403:142–149. 10.1111/nyas.13431 28815614 10.1111/nyas.13431 PMC5664214

[CR136] Schachter AS, Davis KL (2000) Alzheimer’s disease. Dialogues Clin Neurosci 2:91–100 22034442 10.31887/DCNS.2000.2.2/asschachter PMC3181599

[CR137] Sender R, Fuchs S, Milo R (2016) Are We Really vastly outnumbered? Revisiting the ratio of bacterial to host cells in humans. Cell 164:337–340. 10.1016/j.cell.2016.01.013 26824647 10.1016/j.cell.2016.01.013

[CR138] Seo J, Kritskiy O, Watson LA et al (2017) Inhibition of p25/Cdk5 attenuates tauopathy in mouse and iPSC models of frontotemporal dementia. J Neurosci 37:9917–9924. 10.1523/JNEUROSCI.0621-17.2017 28912154 10.1523/JNEUROSCI.0621-17.2017 PMC5637118

[CR139] Shalabi H, Hassan AS, Al-Zahrani FA et al (2023) Intermittent fasting: benefits, side effects, quality of life, and knowledge of the saudi population. Cureus 15:e34722. 10.7759/cureus.34722 36909028 10.7759/cureus.34722 PMC9998115

[CR140] Sharma A, Singh AK (2023) Molecular mechanism of caloric restriction mimetics-mediated neuroprotection of age-related neurodegenerative diseases: an emerging therapeutic approach. Biogerontology 24:679–708. 10.1007/s10522-023-10045-y 37428308 10.1007/s10522-023-10045-y

[CR141] Shintani T, Sakoguchi H, Yoshihara A et al (2017) d-Allulose, a stereoisomer of d-fructose, extends caenorhabditis elegans lifespan through a dietary restriction mechanism: a new candidate dietary restriction mimetic. Biochem Biophys Res Commun 493:1528–1533. 10.1016/j.bbrc.2017.09.147 28965946 10.1016/j.bbrc.2017.09.147

[CR142] Shintani T, Shintani H, Sato M, Ashida H (2023) Calorie restriction mimetic drugs could favorably influence gut microbiota leading to lifespan extension. GeroScience 45:3475–3490. 10.1007/s11357-023-00851-0 37389698 10.1007/s11357-023-00851-0 PMC10643761

[CR143] Silamiķele L, Silamiķelis I, Ustinova M et al (2021) Metformin strongly affects gut microbiome composition in high-fat diet-induced type 2 diabetes mouse model of both sexes. Front Endocrinol 12:626359. 10.3389/fendo.2021.626359 10.3389/fendo.2021.626359 PMC8018580 33815284

[CR144] Silvestro S, Bramanti P, Mazzon E (2021) Role of quercetin in depressive-like behaviors: findings from animal models. Appl Sci 11:7116. 10.3390/app11157116

[CR145] Singh S, Kumar R, Garg G et al (2021) Spermidine, a caloric restriction mimetic, provides neuroprotection against normal and D-galactose-induced oxidative stress and apoptosis through activation of autophagy in male rats during aging. Biogerontology 22:35–47. 10.1007/s10522-020-09900-z 32979155 10.1007/s10522-020-09900-z

[CR146] Singh A, Srivastava P, Verma AK et al (2023) Curcumin displays a potent caloric restriction mimetic effect in an accelerated senescent model of rat. Biol Futur 74:221–229. 10.1007/s42977-023-00170-7 37247086 10.1007/s42977-023-00170-7

[CR147] Smith DL, Orlandella RM, Allison DB, Norian LA (2021) Diabetes medications as potential calorie restriction mimetics—a focus on the alpha-glucosidase inhibitor acarbose. GeroScience 43:1123–1133. 10.1007/s11357-020-00278-x 33006707 10.1007/s11357-020-00278-x PMC8190416

[CR148] Song X, Liu L, Peng S et al (2022) Resveratrol regulates intestinal barrier function in cyclophosphamide-induced immunosuppressed mice. J Sci Food Agric 102:1205–1215. 10.1002/jsfa.11458 34346509 10.1002/jsfa.11458

[CR149] Soni D, Upadhayay S, Dhureja M et al (2024) Crosstalk between gut–brain axis: unveiling the mysteries of gut ROS in progression of Parkinson’s disease. Inflammopharmacol. 10.1007/s10787-024-01510-2 10.1007/s10787-024-01510-2 38992324

[CR150] Steiner P (2019) Brain fuel utilization in the developing brain. Ann Nutr Metab 75(Suppl 1):8–18. 10.1159/000508054 32564020 10.1159/000508054

[CR151] Stephenne X, Foretz M, Taleux N et al (2011) Metformin activates AMP-activated protein kinase in primary human hepatocytes by decreasing cellular energy status. Diabetologia 54:3101–3110. 10.1007/s00125-011-2311-5 21947382 10.1007/s00125-011-2311-5 PMC3210354

[CR152] Strandwitz P (2018) Neurotransmitter modulation by the gut microbiota. Brain Res 1693:128–133. 10.1016/j.brainres.2018.03.015 29903615 10.1016/j.brainres.2018.03.015 PMC6005194

[CR153] Sudo N, Chida Y, Aiba Y et al (2004) Postnatal microbial colonization programs the hypothalamic-pituitary-adrenal system for stress response in mice. J Physiol 558:263–275. 10.1113/jphysiol.2004.063388 15133062 10.1113/jphysiol.2004.063388 PMC1664925

[CR154] Takahara M, Takaki A, Hiraoka S et al (2022) Metformin ameliorates chronic colitis in a mouse model by regulating interferon-γ-producing lamina propria CD4+ T cells through AMPK activation. FASEB J 36:e22139. 10.1096/fj.202100831RR 35064693 10.1096/fj.202100831RR

[CR155] Timmers S, Konings E, Bilet L et al (2011) Calorie restriction-like effects of 30 days of resveratrol supplementation on energy metabolism and metabolic profile in obese humans. Cell Metab 14:612–622. 10.1016/j.cmet.2011.10.002 22055504 10.1016/j.cmet.2011.10.002 PMC3880862

[CR156] Tran SM-S, Mohajeri MH (2021) The Role of gut bacterial metabolites in brain development. Aging Dis Nutr 13:732. 10.3390/nu13030732 10.3390/nu13030732 PMC7996516 33669008

[CR157] Vasileva LV, Savova MS, Amirova KM et al (2020) Caffeic and chlorogenic acids synergistically activate browning program in human adipocytes: implications of AMPK-and PPAR-mediated pathways. Int J Mol Sci 21:9740. 10.3390/ijms21249740 33371201 10.3390/ijms21249740 PMC7766967

[CR158] Verheggen ICM, de Jong JJA, van Boxtel MPJ et al (2020) Increase in blood-brain barrier leakage in healthy, older adults. Geroscience 42:1183–1193. 10.1007/s11357-020-00211-2 32601792 10.1007/s11357-020-00211-2 PMC7394987

[CR159] Wahl D, Solon-Biet SM, Wang Q-P et al (2018) Comparing the effects of low-protein and high-carbohydrate diets and caloric restriction on brain aging in mice. Cell Rep 25:2234-2243.e6. 10.1016/j.celrep.2018.10.070 30463018 10.1016/j.celrep.2018.10.070 PMC6296764

[CR160] Wakade C, Chong R, Bradley E, Morgan JC (2015) Low-dose niacin supplementation modulates GPR109A, niacin index and ameliorates Parkinson’s disease symptoms without side effects. Clin Case Rep 3:635–637. 10.1002/ccr3.232 26273459 10.1002/ccr3.232 PMC4527813

[CR161] Wang H-X, Wang Y-P (2016) Gut microbiota-brain axis. Chin Med J 129:2373–2380. 10.4103/0366-6999.190667 27647198 10.4103/0366-6999.190667 PMC5040025

[CR162] Wang N, Han Q, Wang G et al (2016) Resveratrol protects oxidative stress-induced intestinal epithelial barrier dysfunction by upregulating heme oxygenase-1 expression. Dig Dis Sci 61:2522–2534. 10.1007/s10620-016-4184-4 27146412 10.1007/s10620-016-4184-4

[CR163] Wang N, Luo Z, Jin M et al (2019) Exploration of age-related mitochondrial dysfunction and the anti-aging effects of resveratrol in zebrafish retina. Aging 11:3117–3137. 10.18632/aging.101966 31105084 10.18632/aging.101966 PMC6555466

[CR164] Wenzel TJ, Gates EJ, Ranger AL, Klegeris A (2020) Short-chain fatty acids (SCFAs) alone or in combination regulate select immune functions of microglia-like cells. Mol Cell Neurosci 105:103493. 10.1016/j.mcn.2020.103493 32333962 10.1016/j.mcn.2020.103493

[CR165] Wilson DM, Cookson MR, Van Den Bosch L et al (2023) Hallmarks of neurodegenerative diseases. Cell 186:693–714. 10.1016/j.cell.2022.12.032 36803602 10.1016/j.cell.2022.12.032

[CR166] Wirth M, Benson G, Schwarz C et al (2018) The effect of spermidine on memory performance in older adults at risk for dementia: a randomized controlled trial. Cortex 109:181–188. 10.1016/j.cortex.2018.09.014 30388439 10.1016/j.cortex.2018.09.014

[CR167] Wu P, Shen Q, Dong S et al (2008) Calorie restriction ameliorates neurodegenerative phenotypes in forebrain-specific presenilin-1 and presenilin-2 double knockout mice. Neurobiol Aging 29:1502–1511. 10.1016/j.neurobiolaging.2007.03.028 17499883 10.1016/j.neurobiolaging.2007.03.028

[CR168] Yang L, Jiang Y, Shi L et al (2020a) AMPK: Potential therapeutic target for Alzheimer’s disease. Curr Protein Pept Sci 21:66–77. 10.2174/1389203720666190819142746 31424367 10.2174/1389203720666190819142746

[CR169] Yang LL, Millischer V, Rodin S et al (2020b) Enteric short-chain fatty acids promote proliferation of human neural progenitor cells. J Neurochem 154:635–646. 10.1111/jnc.14928 31784978 10.1111/jnc.14928

[CR170] Yang J-Y, Liu M-J, Lv L et al (2022) Metformin alleviates irradiation-induced intestinal injury by activation of FXR in intestinal epithelia. Front Microbiol 13:932294. 10.3389/fmicb.2022.932294 36312920 10.3389/fmicb.2022.932294 PMC9608595

[CR171] Zhang Q, Xiao X, Li M et al (2013) Acarbose reduces blood glucose by activating miR-10a-5p and miR-664 in diabetic rats. PLoS ONE 8:e79697. 10.1371/journal.pone.0079697 24260283 10.1371/journal.pone.0079697 PMC3832586

[CR172] Zhang X-W, Chen J-Y, Ouyang D, Lu J-H (2020) Quercetin in animal models of Alzheimer’s disease: a systematic review of preclinical studies. Int J Mol Sci 21:493. 10.3390/ijms21020493 31941000 10.3390/ijms21020493 PMC7014205

[CR173] Zhang H, Chen Y, Wang Z et al (2022) Implications of gut microbiota in neurodegenerative diseases. Front Immunol. 10.3389/fimmu.2022.785644 35237258 10.3389/fimmu.2022.785644 PMC8882587

[CR174] Zhao J, Zhao F, Yuan J et al (2023) Gut microbiota metabolites, redox status, and the related regulatory effects of probiotics. Heliyon 9:e21431. 10.1016/j.heliyon.2023.e21431 38027795 10.1016/j.heliyon.2023.e21431 PMC10643359

[CR175] Zhu M, Liu X, Ye Y et al (2022) Gut microbiota: a novel therapeutic target for Parkinson’s disease. Front Immunol 13:937555. 10.3389/fimmu.2022.937555 35812394 10.3389/fimmu.2022.937555 PMC9263276

[CR176] Zhu X, Shen J, Feng S et al (2023) Akkermansia muciniphila, which is enriched in the gut microbiota by metformin, improves cognitive function in aged mice by reducing the proinflammatory cytokine interleukin-6. Microbiome 11:120. 10.1186/s40168-023-01567-1 37254162 10.1186/s40168-023-01567-1 PMC10228018

